# A secreted C-type lectin in Periplaneta americana functioning in antibacterial activity, innate immune signaling and leg regeneration

**DOI:** 10.3389/fimmu.2025.1730116

**Published:** 2026-01-20

**Authors:** Xiaoxuan Liu, Nan Sun, Shuang Geng, Shuqi Xian, Xiaojuan Wu, Ying Huang, Yechun Pei

**Affiliations:** 1 School of Life and Health Sciences, Hainan Province Key Laboratory of One Health, Collaborative Innovation Center of Life and Health, Hainan University, Haikou, Hainan, China; 2 School of Tropical Agriculture and Forestry, Hainan University, Haikou, China; 3 School of life science, Shanxi University, Datong, China; 4 Hainan International One Health Institute, Hainan University, Haikou, Hainan, China

**Keywords:** C-type lectins, immune response, PaSCLec, periplaneta americana, regeneration

## Abstract

**Introduction:**

C-type lectins are important pattern-recognition receptors that play essential roles in innate immune responses by recognizing pathogen-associated molecular patterns. However, their biological functions in *Periplaneta americana* have not been systematically investigated.

**Methods:**

Two PaSCLec isoforms predicted by RNA-seq, designated PaSCL-Ad and PaSCL-Reg, were identified using Rapid Amplification of cDNA Ends (RACE) and Nanopore sequencing. Tissue-specific expression and inducible expression following bacterial challenge were analyzed. Recombinant PaSCL-Ad and PaSCL-Reg proteins were produced to examine their binding activities to microbial polysaccharides, bacterial agglutination, antimicrobial effects, and membrane-disruptive activity. Hemocyte phagocytosis was evaluated by immunocytochemical analysis. RNA interference was employed to assess the role of PaSCLec in immune regulation and leg regeneration, followed by transcriptional analyses.

**Results and Discussion:**

Total PaSCLec (PaSCL-Ad and PaSCL-Reg) was most highly expressed in the hemolymph and was significantly upregulated after challenge with *Escherichia coli* and Staphylococcus aureus. Both recombinant proteins bound lipopolysaccharides, peptidoglycan, mannan, and β-glucan in a dose-dependent manner, and agglutinated Gram-positive (*Staphylococcus aureus*, *Bacillus subtilis*) and Gram-negative (*Escherichia coli*, *Salmonella Typhimurium*) bacteria in a Ca^2+^-dependent manner. rPaSCL-Ad inhibited the growth of all tested bacteria and potentially compromised the membrane integrity of *E. coli* in a Ca^2+^-independent manner. Both recombinant proteins enhanced hemocyte phagocytic activity by promoting bacteria–hemocyte interactions. Knockdown of *PaSCLec* reduced the expression of multiple antimicrobial peptides and transcription factors associated with the Toll, IMD, and JAK/STAT signaling pathways; these effects were partially rescued by recombinant protein supplementation. In addition, *PaSCLec* knockdown impaired leg regeneration. qRT-PCR suggested an association between *PaSCLec* activity and JAK/STAT-related genes during regeneration. These findings demonstrate that *PaSCLec* is a multifunctional secreted lectin involved in microbial recognition, immune effector regulation, and leg regeneration in *P. americana*, although the underlying molecular mechanisms require further investigation.

## 1 Introduction

C-type lectins (CTLs), a major class of pattern recognition receptors (PRRs), play essential roles in innate immunity (1, 2) and occur either as transmembrane receptors or as soluble secreted proteins (3). Previous studies have demonstrated that CTLs participate in both immune responses and developmental processes in invertebrates. (4, 5). In *Tribolium castaneum*, multiple CTLs have been identified and shown to contribute to immune responses and development (6–9). In *Drosophila melanogaster*, DL2 and DL3 can agglutinate *E. coli* and promote encapsulation and melanization *in vitro* (10, 11). In *Rhynchophorus ferrugineus*, RfCTL27 recognizes Gram-negative bacteria and induces antimicrobial peptide expression to eliminate pathogens (12). In *Ostrinia furnacalis*, IML-10 promotes hemocyte aggregation by binding directly to hemocyte surfaces, thereby facilitating encapsulation (13). In addition, CLEC3A activates the PI3K–AKT pathway to accelerate cell proliferation. (14). *In vitro* analyses have also shown that Collectin-11, a soluble C-type lectin, activates EGFR signaling and directly promotes murine melanoma cell proliferation (15). Collectively, these findings indicate that CTLs are critical regulators of both immunity and development.

Insects are invertebrates of substantial economic, ecological, pathological, and medicinal importance (16–18). *Periplaneta americana* is widely used as a raw material in traditional Chinese medicine (19). Extracts of *P. americana* exhibit strong regenerative properties (20–22), which may be associated with its remarkable leg regeneration capabilities (23). Previous studies have shown that CTLs from *P. americana* act as opsonins that mediate immune responses (24–26). Regenectin localizes near newly formed epidermal cells, is secreted into the regenerating leg saccule, and assembles around myoblasts to facilitate the in site formation of leg muscle fibers (27). A potential link exists between immune responses and leg regeneration, and C-type lectins may play a central role in this process. Transcriptome analyses previously identified two *PaSCLec* isoforms, PaSCL-Reg and PaSCL-Ad, both of which are highly expressed during leg regeneration in P. americana (28). However, the biological function of *PaSCLec* remain unclear.

Here, we investigated whether the secreted C-type lectin PaSCLec contributes to immune regulation and leg regeneration. We found that bacterial challenge significantly increased the mRNA levels of total *PaSCLec* (including *PaSCL-Reg* and *PaSCL-Ad*) in hemocytes. In addition, both PaSCL-Reg and PaSCL-Ad exhibited bacterial-binding activity, agglutinating capacity, and antibacterial properties. Furthermore, *PaSCLec* knockdown impaired leg regeneration, likely by modulating the expression of unpaired. These findings enhance our understanding of the roles of secreted C-type lectins in immune responses and leg regeneration, and further expand the functional diversity attributed to CTLs.

## 2 Materials and methods

### 2.1 Insects

All Cockroaches were provided by American cockroach breeding farm in Chizhou city, Anhui province, China. The colony was maintained at 27°C with a relative humidity of 70-80% in breathable plastic box, and the animals received lab mice diet and water. To obtain pools of synchronized animals, the oothecae were hatched uniformly and molt numbers were recorded. Newly 6^th^ instar nymphs were selected from the colony and placed in separate plastic boxes, supplied with mice diet and water. The experimental cockroaches were anesthetized with ice for sample collection.

### 2.2 Bioinformatics analysis of *PaSCLec*

The full-length transcript sequence of *PaSCLec* was obtained from cockroach leg by transcriptomic sequencing. Transcript sequence was used for primer design to obtain full-length cDNAs by Rapid Amplification of cDNA Ends (RACE) using the HiScript-TS 5’/3’ RACE Kit (Vazyme, China). A cDNA copy of PaSCLec was obtained using the following primers: 3′RACE (5′- ATGGGCGCTGGAAGCTGTATACCGG-3′) and 5′RACE (5′ -AGGGGTTCATGCGGCTTATGA-3′). The sequence of *PaSCLec* was subjected to nanopore sequencing by Beijing Tsingke Biotech Co., Ltd. The full-length cDNA sequence of *PaSCL-Reg* and *PaSCL-Ad* were amplified by RT-PCR using corresponding primers and re-sequenced for confirmation (**Supplementary Table S1**). Homology analysis of PaSCL-Reg and PaSCL-AD amino acid sequence were performed using BLAST (http://www.ncbi.nlm.nih.gov/). Multiple sequences alignment analysis was performed using the DNAMAN. The characterizations of protein were predicted on the ExPASY server (http://www.expasy.org/). SignalP 5.0 server was used to predict the signal peptide (https://services.healthtech.dtu.dk/service.php?SignalP-5.0). Domain architecture prediction of the proteins was performed using SMART (http://smart.embl-heidelberg.de/). MEGA 5 was used for phylogenetic analysis. The modeling structures were generated by predicted by using Alphafold3 server (https://alphafoldserver.com/).

### 2.3 Preparation of rPaSCL-Reg and rPaSCL-Ad

The codon-optimized sequences of rPaSCL-Reg and rPaSCL-Ad were synthesized *de novo* by Beijing Tsingke Biotech Co., Ltd and subsequently cloned into the plasmid (pET28a) before transformation into BL21 (DE3) cells to generate recombinant plasmids. Positive clones underwent PCR screening with primers T7-F and T7-R, followed by sequence confirmation. A single colony of transformed BL21 (DE3) cells was induced to express the recombinant protein with 0.1 mM IPTG at 28°C for 6 h. The resulting rPaSCL-Reg and rPaSCL-Ad were purified using a Ni column (Qiagen, Germany) and underwent desalination via Amicon^®^ Ultra (Merck, Germany), followed by analysis using 10% SDS-PAGE and Coomassie brilliant blue R-250 staining. Recombinant protein concentration was determined using BCA protein Assay Kit (Solarbio, China).

### 2.4 Western blot analysis

Following SDS-PAGE separation, the purified proteins were transferred to a nitrocellulose membrane (Pall, America). The membranes underwent blocking for 2 h with 5% non-fat milk in 1×TBST (10 mM Tris·HCl, 150 mM NaCl, 0.1% Tween, pH 7.2-7.6 in 25°C). After washing, the membrane was incubated with 1/5000 primary antibody (Rabbit anti-His-tag antibody, Abclonal, China) in TBST containing 5% non-fat milk at 4°C for 12 h. The washed membrane was then incubated with a secondary antibody (HRP-conjugated Goat Anti-Rabbit IgG, BBI, China) at 37°C for 2 h. The Western blot analysis was performed on UVP ChemStudio815 using an enhanced Chemiluminescence Substrate Kit (Yuanye, China).

### 2.5 Recombinant protein binding assay

Bacterial cultures were grown overnight and harvested by centrifugation at 8000 r for 10 min. The bacteria underwent three washing cycles with TBS (10 mM Tris·HCl, 150 mM NaCl, pH 7.2-7.6 in 25°C) and were resuspended in TBS to achieve an OD600 of 1.0. The bacterial suspension (100 μL) in TBS was incubated with purified rPaSCL-Reg and rPaSCL-Ad (50 μg) for 60 min at room temperature under mild rotation. Following incubation, the bacteria were washed four times with TBS. The final bacterial suspension underwent 10% SDS-PAGE and Western blot analysis. Rabbit anti-His-tag antibody (Abclonal, China, 1:5000) served as the primary antibody, while HRP-conjugated Goat Anti-Rabbit IgG (BBI, China, 1:10, 000) functioned as the secondary antibody, respectively.

An Elisa was employed to assess the sugar binding specificity of rPaSCL-Reg and rPaSCL-Ad. LPS from *E. coli* and PGN from *S. aureus* were selected for the assay. 96-well ELISA Plates were coated with 10 μg of polysaccharide and incubated at 4°C overnight. After five TBS washes, the microplates underwent blocking with BSA (1 mg/mL, 200 μL) at 37°C for 2 h, followed by TBS washing. Purified rPaSCL-Reg and rPaSCL-Ad (final concentration 0–80 μg/mL in TBS with 0.1 mg/mL BSA) were added to each coated well and incubated at 37°C for 2 h. The plate underwent five TBS washes. Subsequently, Rabbit anti-His-tag antibody (1:2000) was added (100 μL per well) and incubated at 37˚C for 2 h; 100 μL of HRP-conjugated Goat Anti-Rabbit IgG (1:5000) was introduced to each well at 37°C for 1 h. Following five TBS washes, color development occurred with TMB (BBI, China) at room temperature for 30 min. The OD value was measured at 450 nm. Each binding assay was performed in triplicate.

### 2.6 Agglutination test

Gram-positive bacteria (*Staphylococcus aureus* and *Bacillus subtilis*) and Gram-negative bacteria (*Escherichia coli* and *Salmonella Typhimurium*) were selected to evaluate the bacterial agglutination properties of rPaSCL-Reg and rPaSCL-Ad. A volume of 25 μL rPaSCL-Reg and rPaSCL-Ad (100 μg/mL) was incubated with an equal volume of bacterial suspension (1×10^8^ CFU/mL) at room temperature (~25°C) for 1 h, both with and without 10 mmol/L CaCl_2_. rBSA in TBS and TBS+Ca^2+^ (10 mM Tris·HCl, 150 mM NaCl, 10 mM CaCl_2_, pH 7.2-7.6 in 25°C) served as negative controls. Following incubation, agglutination was observed and documented using a fluorescence microscope (Nikon, Japan).

### 2.7 Antibacterial activity assays

A total of 100 μg of each protein was incubated with a bacterial suspension (1 × 10^5^ CFU/mL) in fresh LB (Tryptone 10 g/L, Yeast Extract 5g/L, NaCl 10g/L) in a 96-well culture plate. CaCl_2_ was added to a final concentration of 10 mM, with each well containing a total volume of 200 μL. The plate was incubated at 37 °C for 24 h. Bacterial growth was assessed by measuring the absorbance at 600 nm using a microplate reader. Each assay was performed in triplicate wells per protein and repeated in three independent experiments. rBSA served as a negative control. Data are presented as mean ± standard deviation (SD), and statistical significance was evaluated using student t-test.

### 2.8 Immunocytochemical analysis

Hemolymph extracted from *P. americana* was fixed using 200 μL of anticoagulant mixture (62 mM NaCl, 100 mM glucose, 10 mM EDTA, 30 mM Sodium citrate, 26 mM citric acid) and 4% paraformaldehyde, followed by centrifugation at 600 g for 10 min at 4°C. The isolated hemocytes were placed on poly-L-lysine-Prep slides (BBI, China) to facilitate cell adhesion during microscopic analysis, washed with TBS, and blocked with 5% BSA at 37°C for 30 min. Following TBS washing, the hemocytes were incubated with 10 μg rPaSCL-Reg or rPaSCL-Ad at 4°C for 12 h. The samples were then washed with TBS and incubated with 5% anti-His-tag antibody (1:1000 in 5% BSA) at room temperature for 2 h, followed by TBS washing and incubation with Alexa Fluor 488-conjugated Goat anti-rabbit IgG (Abclonal, China, 1:1000 in 5% BSA) for 1 h at 37°C in darkness. After six washes, the hemocytes were treated with 2-(4-Amidinophenyl)-6-indolecarbamidine dihydrochloride (DAPI, Beyotime, China) for 10 min at room temperature and washed six times. Fluorescence was examined using a laser scanning confocal microscope (LSCM) (Nikon, Japan). Nis-ElemeViewer software was utilized to assess the binding capability of rPaSCL-Reg and rPaSCL-Ad to hemolymph membranes.

### 2.9 Phagocytosis assay

To assess the phagocytic activity of hemocytes, Hemolymph was collected with anticoagulant and centrifuged at 600 g for 10 min at 4 °C to isolate hemocytes. Hemocytes were gently washed twice with TBS and resuspended. *Escherichia coli* was cultured overnight, harvested by centrifugation at 5000 rpm for 10 min, washed three times with TBS, and resuspended. Bacteria were labeled with 0.1 mg/mL FITC which dissolved in DMSO for 2 h in the dark, washed three times with TBS to remove excess dye, and finally resuspended in TBS at an OD600 = 1. For the phagocytosis assay, 100 µL of hemocyte suspension (1×10^6^ cells/mL) was added to each well of a confocal culture dish containing culture medium. The treatment group received 10 µg of rPaSCL-Reg and rPaSCL-Ad, whereas the control group received rBSA, with three replicates per group. Cells were incubated at 28 °C for 1 h. Subsequently, FITC-labeled bacteria were added at a 1:10 ratio of hemocytes to bacteria. Hemocytes were fixed with 4% paraformaldehyde for 30 min, washed three times with PBS, and 10 µL of the cell-bacteria mixture was placed onto a microscope slide for 10 min sedimentation in a humid chamber. Phagocytosis was observed under a LSCM (Nikon, Japan), and images were captured. For each treatment, at least 150 hemocytes from randomly selected fields were counted to calculate the phagocytic rate.

### 2.10 Scanning electron microscopy

Morphological changes in recombinant protein-bound bacteria were examined using field-emission scanning electron microscopy (Verios G4 UC, Thermo Fisher Scientific, America). Bacterial suspensions (1×10^8^ CFU/mL) were incubated with rPaSCL-Reg and rPaSCL-Ad at 2 mg/mL in TBS and TBS+Ca^2+^ (TBS, 10 mM CaCl_2_) at 37°C for 2 h, using rBSA protein (2 mg/mL) as the negative control. After incubation, cells underwent fixation with 2.5% (v/v) glutaraldehyde in 0.1 M Phosphate buffer at 4°C for 12 h, followed by gradual dehydration using increasing concentrations of ethanol (30%, 50%, 70%, 80%, 90%, and 100%) for 20 min at each step. The samples were subsequently dried, gold coated, and analyzed using field-emission scanning electron microscopy.

### 2.11 Real-time quantitative PCR

For gene expression analyses, 10-fold diluted cDNA served as templates for qRT-PCR. Reactions were performed in triplicate using ChamQ Universal SYBR qPCR Master Mix (Vazyme, Q711). The relative expression levels were calculated using the 2^-ΔΔCt^ method and there were three biological replicates and three technical replicates for each sample. *Actin* was chosen as a reference gene for qRT-PCR analysis (20). The primers used for qRT-PCR and the amplification efficiency (29) of each primer are shown in **Supplementary Table S1**.

### 2.12 Double-stranded RNA treatment

Double-stranded RNA (dsRNA) was synthesized using the T7 RiboMAX Express RNA interference (RNAi) System (Promega, P1700). Following purification, dsRNA was prepared at a concentration of 2 μg/μL, and 2μg was microinjected into the coxa of each nymph. To sustain RNAi efficiency throughout the regeneration process, injections were repeated every 7 days (15 nymphs per replicate, three biological replicates). The dsRNA-synthesizing primer sequences are detailed in **Supplementary Table S1**. A dsMock targeting a clone vector sequence was used as the negative control. For the analysis of differentially expressed genes following RNAi of target genes, dsRNA was performed together with amputation, and the coxa and trochanter at 7 days post-amputation (dpa) were harvested.

## 3 Results

### 3.1 Cloning and sequence analysis of PaSCLec

**Figure 1:**
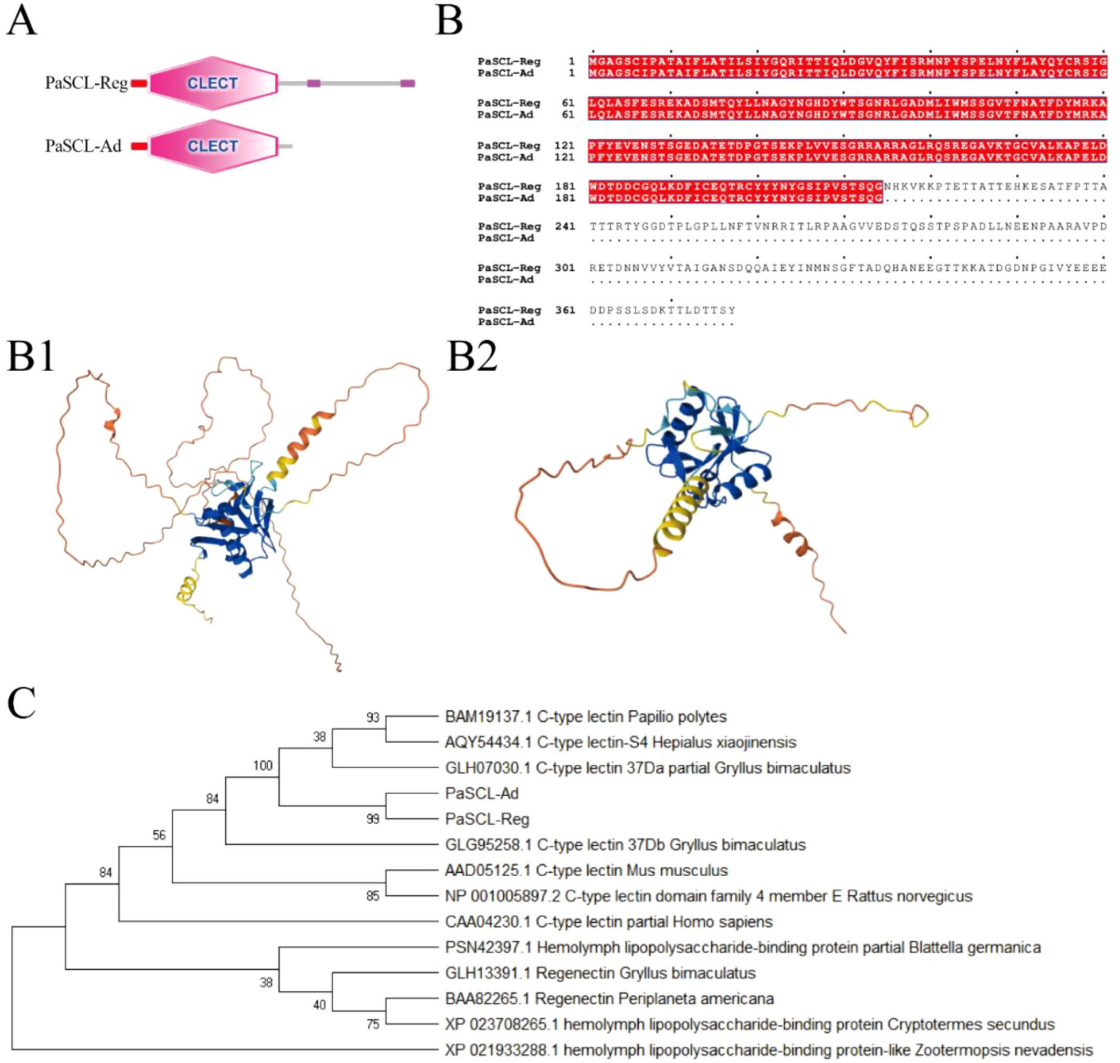
Sequence analysis of PaSCL-Reg and PaSCL-Ad **(A)** Red square and pink hexagon represent signal peptide and CTLD domain. **(B)** Multiple alignments of the PaSCL-Reg with PaSCL-Ad. The structure of the PaSCL-Reg (B1) and PaSCL-Ad (B2) were analyzed using the online Alphafold3 server. **(C)** Phylogenetic analysis of PaSCL-Reg and PaSCL-Ad with other CTLs from various species. All amino acid sequences were collected from NCBI, and the tree was constructed with MEGA X software.

Through transcriptome analysis of leg regeneration in *Periplaneta americana*, a unique secreted C-type lectin (*PaSCLec*) with high expression was identified. Notably, an alternate donor site event in the transcript of *PaSCLec* generating a frameshift mutation revealed two distinct isoforms (designated PaSCL-Ad and PaSCL-Reg) (**Supplementary Figure S1**). Using 5′/3′ RACE and Nanopore sequencing, we found that transcripts corresponding to PaSCL-Ad constituted approximately 70–80% of the total isoform population (Primer 3′RACE: 79.06%; Primer 5′RACE: 75.46%). Therefore, the transcript of *PaSCL-Ad* was considered the main transcript. Sequence analysis showed the ORF of PaSCL-Ad (accession no. XP_069685827.1) encodes 214 amino acids with a molecular weight (MW) of 23.9 kDa and an isoelectric point of 4.97, while the ORF of PaSCL-Reg (accession no. XP_069685825.1) encodes 377 amino acids with a molecular weight (MW) of 41.3 kDa and an isoelectric point of 4.65. Both PaSCL-Ad and PaSCL-Reg proteins contain a 22-amino acid signal peptide and a 173-amino acid C-type lectin-like domain (**Figure 1A**). The amino acid sequence analysis indicates PaSCL-Reg contains 163 additional amino acids compared to PaSCL-Ad at its C-terminal (**Figure 1B**). A comparison of protein structure reveled that the overall architecture of PaSCL-Ad is relatively compact, with a well-defined core domain (**Figure 1B1**). In contrast, although PaSCL-Reg also possesses a recognizable core structure, it appears more loosely organized, suggesting that its overall structural stability may be lower (**Figure 1B2**). Phylogenetic analysis of PaSCL-Ad, PaSCL-Reg and other CTLs from invertebrate and vertebrate species revealed that, unlike Regenectin, PaSCL-Ad and PaSCL-Reg cluster with CTLs from *Gryllus bimaculatus*, which also possesses leg regeneration capability (**Figure 1C**).

### 3.2 Expression profile of total PaSCLec

**Figure 2:**
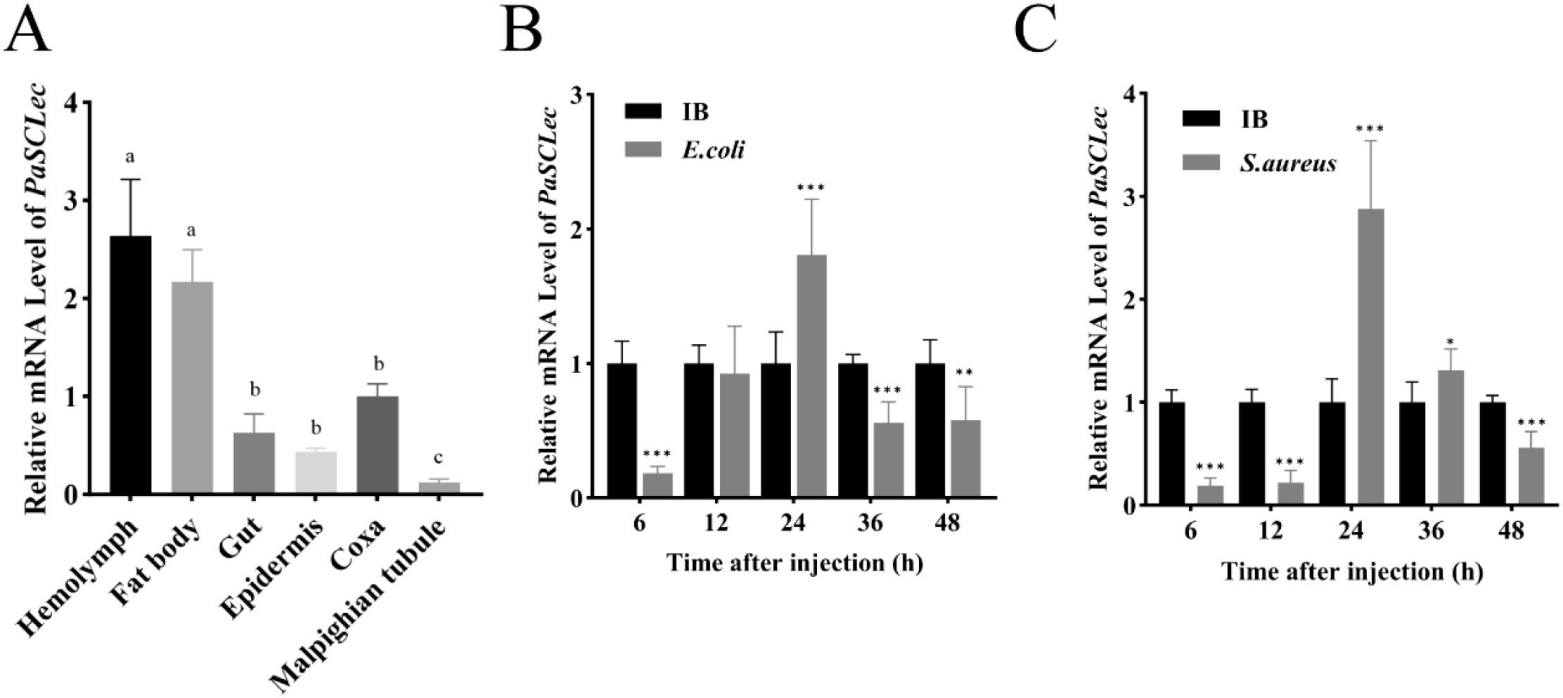
Expression profile of total *PaSCLec*. **(A)** Relative mRNA levels of *PaSCLec* in different tissues. Relative mRNA levels of *PaSCLec* in hemolymph after *E. coli***(B)** and *S. aureus***(C)** treatment. The results are the mean and standard errors of three biological replicates. Different letters on the error bar indicate statistically significant differences at *p <* 0.05 level (ANOVA in association with Tukey’s HSD test). Asterisk indicates significant differences compared with values of the control (student’s t test, *p < 0.05, **p < 0.01, ***p < 0.001). ANOVA, one-way analysis of variance; HSD, honestly significant difference; IB: injection buffer (control); mRNA, messenger ribonucleic acid.

Tissue expression profiling revealed that total *PaSCLec* exhibited highest expression in the hemolymph, followed by fat body, coxa and gut (**Figure 2A**). To investigate the potential role o*f PaSCLec* in innate immunity, sixth instar *P. americana* were subjected to bacterial injection to induce a systemic immune challenge. Compared with the control group, the mRNA levels of *PaSCLec* in hemolymph were significantly upregulated at 24 h post-injection with either *E. coli* (**Figure 2B**) or *S. aureus* (**Figure 2C**). These results suggest *PaSCLec* is involved in the immunoregulatory network of *P. americana*.

### 3.3 Expression and purification of rPaSCL-Ad and rPaSCL-Reg

**Figure 3:**
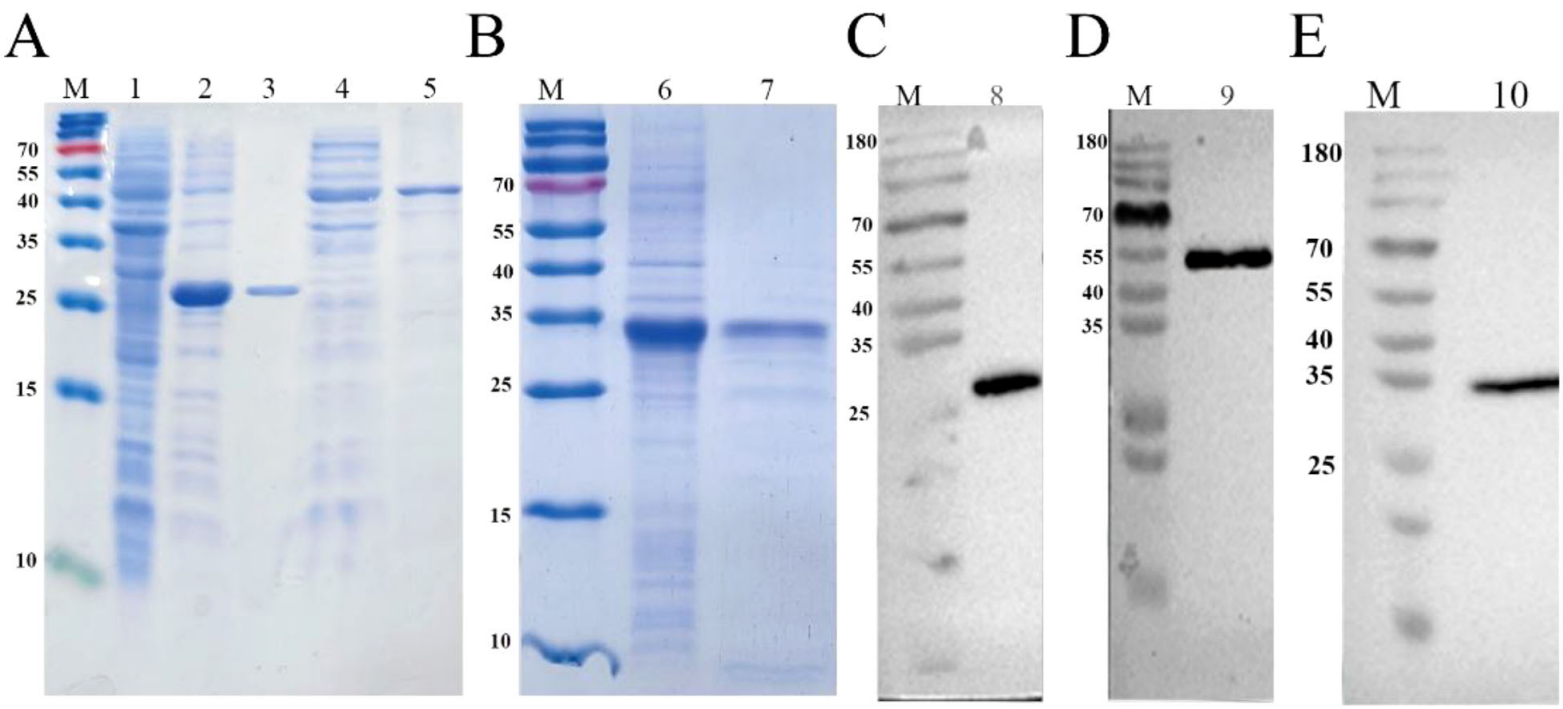
The expression and purification of rPaSCL-Ad, rPaSCL-Reg and rRegenectin. rPaSCL-Reg, rPaSCL-Ad and rRegenectin were detected by sodium dodecyl sulfate polyacrylamide gel electrophoresis (SDS–PAGE) **(A, B)** and Western blot **(C-E)**. Lane M: molecular marker; Lane 1: crude supernatant protein extracts of bacteria with pET-28a; Lane 2, lane 4 and lane 6, crude supernatant protein extracts of bacteria with pET-28a-PaSCL-Ad, pET-28a-PaSCL-Reg and pET-28a-PaSCL-Regenectin proteins; Lane 3, lane 5 and lane 7, purified pET-28a-PaSCL-Ad, pET-28a-PaSCL-Reg and pET-28a-PaSCL-Regenectin proteins; Lane 8, lane9 and lane 10, western blot based on the purified pET-28a-PaSCL-Ad, pET-28a-PaSCL-Reg and pET-28a-PaSCL-Regenectin proteins.

The alternative splicing variants rPaSCL-Ad and rPaSCL-Reg were expressed using a prokaryotic expression system to assess their biological activities and purified using the Ni-NTA Agarose (QIAGEN, Germany). The purification of rPaSCL-Ad and rPaSCL-Reg was verified by sodium dodecyl sulfate polyacrylamide gel electrophoresis (SDS-PAGE) (**Figure 3A**). To assess the immune functions of rPaSCL-Ad and rPaSCL-Reg, another *P. americana* C-type lectin, Regenectin, which is expressed during leg regeneration but has no reported immune activity, was also purified as a comparative control (**Figure 3B**).The purified rPaSCL-Ad, rPaSCL-Reg and Regenectin were analyzed using Western blot (**Figure 3C, D, E**), revealing apparent molecular weights of approximately ~26 kDa and ~40 kDa for rPaSCL-Ad and rPaSCL-Reg respectively, which correspond precisely to their predicted sizes.

### 3.4 Bacterial binding and agglutinating activity of rPaSCL-Ad and rPaSCL-Reg

**Figure 4:**
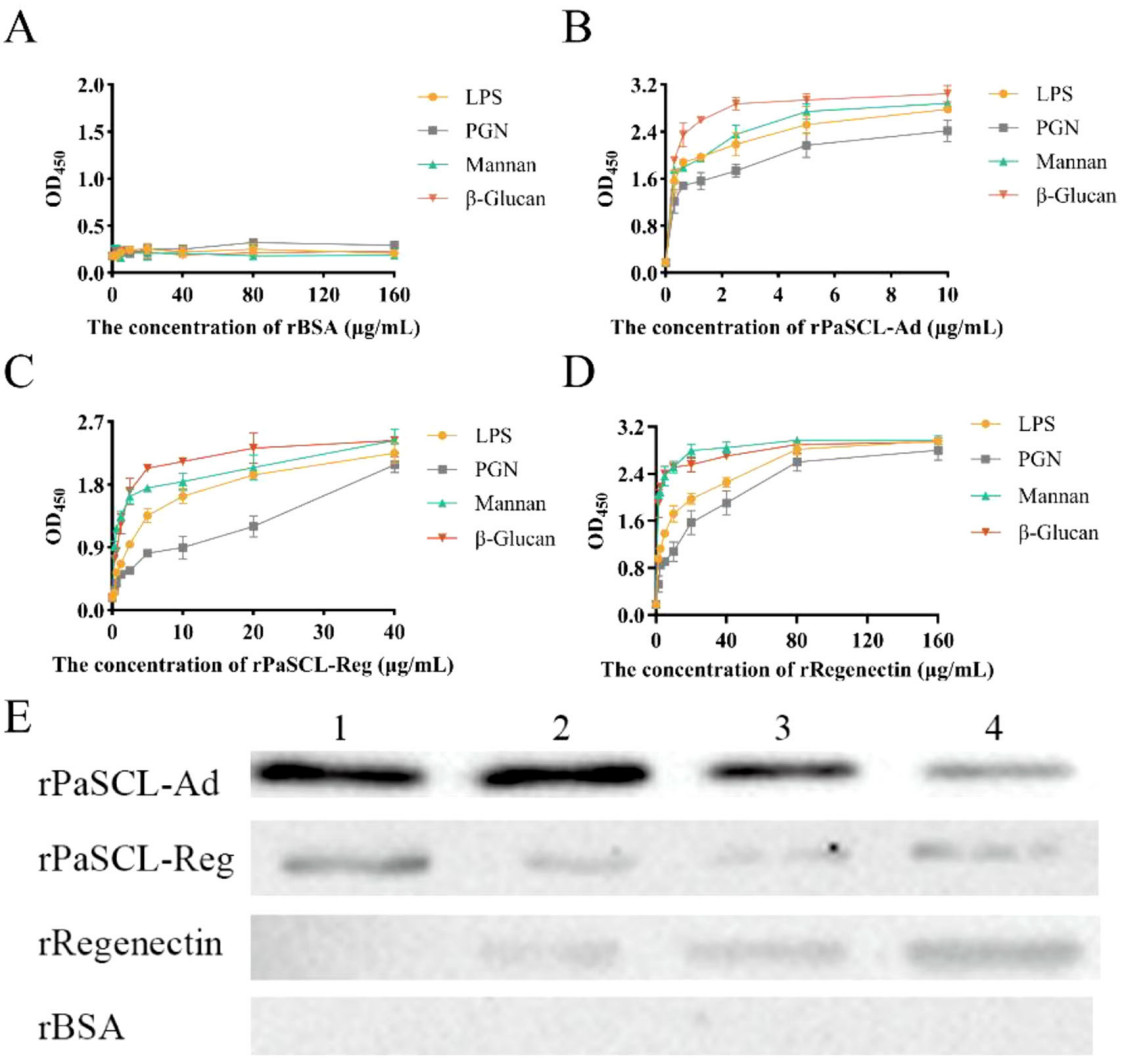
Binding affinity of rPaSCL-Ad, rPaSCL-Reg and rRegenectin. Binding of rBSA **(A)**, rPaSCL-Ad **(B)**, rPaSCL-Reg **(C)** and rRegenectin **(D)** to polysaccharide, analyzed by ELISA. ELISA, enzyme-linked immunosorbent assay; LPS, lipopolysaccharide; PGN, peptidoglycan. Binding of rPaSCL-Ad, rPaSCL-Reg and rRegenectin to bacteria (*E. coli, S. aureus, B. subtilis* and *S. typhimurium*) was detected by Western blot **(E)**. Line 1: *E. coli*; Line 2: *S. aureus*; Line 3: *S. typhimurium*; Line 4: *B. subtilis.*.

C-type lectin exhibits pathogen recognition functions through binding to the Pathogen-Associated Molecular Patterns (PAMPs). Direct binding analysis using enzyme-linked immunosorbent assay (ELISA) demonstrated that rBSA had no binding ability (**Figure 4A**) and rPaSCL-Ad and rPaSCL-Reg bound to lipopolysaccharide (LPS) from *E. coli* and peptidoglycan (PGN) from *S. aureus*, with binding ability showing dose dependence (**Figures 4B, C**). Additionally, rRegenectin displayed lower binding capacity compared to rPaSCL-Ad and rPaSCL-Reg at equivalent concentrations (**Figure 4D**). Furthermore, rPaSCL-Ad demonstrated binding capability to Mannan and β-Glucan at low doses (PAMPs predominantly found in fungal cell walls). Western blotting analysis confirmed the binding affinity of rPaSCL-Ad and rPaSCL-Reg to bacteria. The results indicated that rPaSCL-Ad exhibited superior binding capacity for microorganisms including GP bacteria *S. aureus*, *Bacillus subtilis*, and GN bacteria *Salmonella Typhimurium* and *E. coli* (**Figure 4E**).

**Figure 5:**
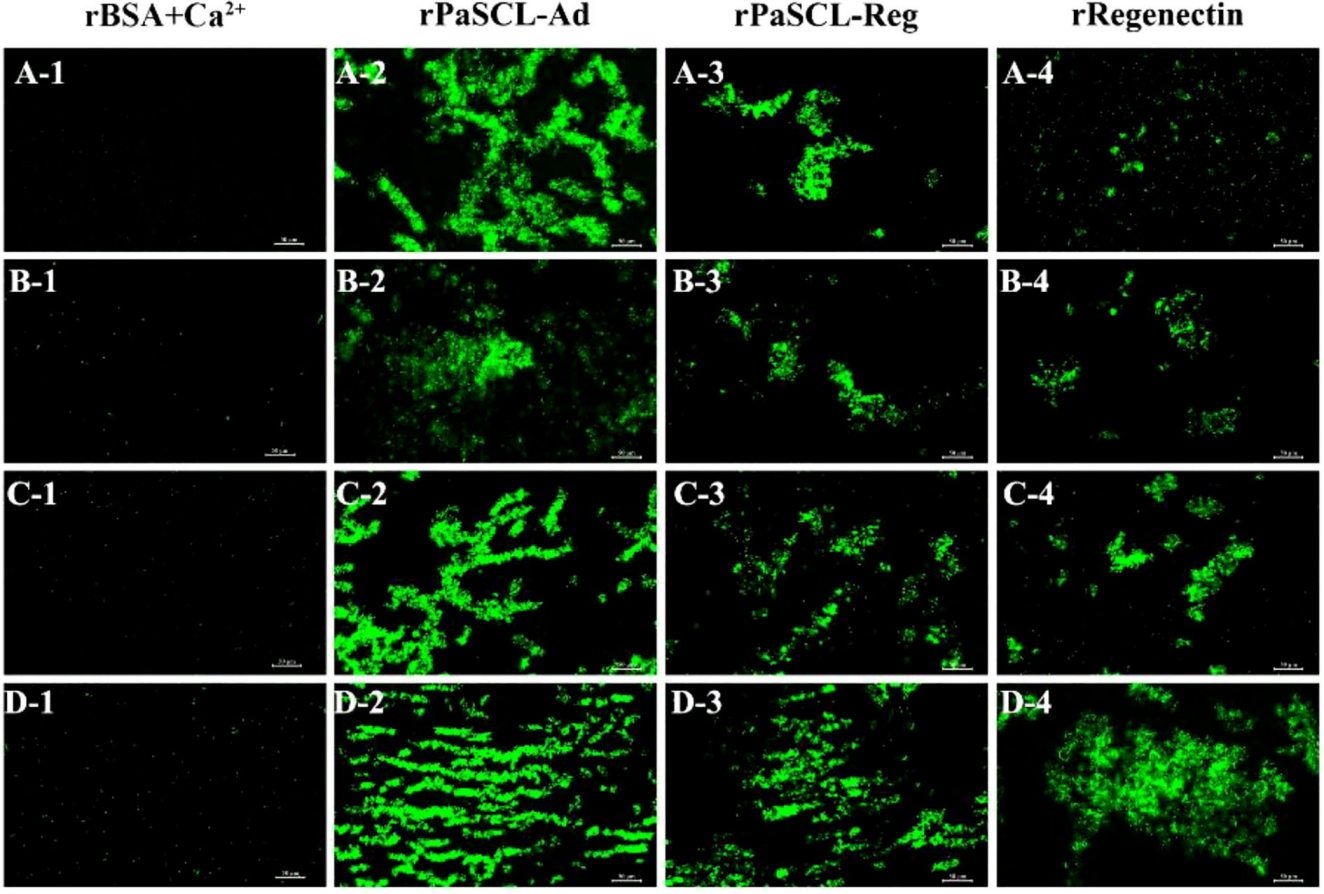
Agglutination activities of rPaSCL-Ad, rPaSCL-Reg and rRegenectin with 10mM Ca^2+^ (recombinant proteins+bacteria). *E. coli***(A)**, *S. aureus***(B)**, *S. typhimurium***(C)***B. subtilis***(D)**. The corresponding control groups are shown in **Supplementary Figure S2**.

The agglutination activity of all recombinant proteins was evaluated using FITC method across various bacteria. All recombinant proteins demonstrated agglutination activity toward bacteria in the presence of Ca^2+^ (**Figure 5**), except for the control rBSA (**Figure 5A**). rPaSCL-Ad demonstrated the strongest agglutination activity (**Figure 5B**). Both rPaSCL-Reg and rRegenectin exhibited comparatively weaker agglutination, with rRegenectin showing almost no detectable activity toward *E. coli* (**Figures 5C, D**); however, no agglutinating activity was observed toward any bacteria without Ca^2+^ (**Supplementary Figure S2**), indicating Ca^2+^-dependent agglutinating activity.

### 3.5 rPaSCL-Ad exhibits bacterial antibacterial activity and destroy the membrane integrity

**Figure 6:**
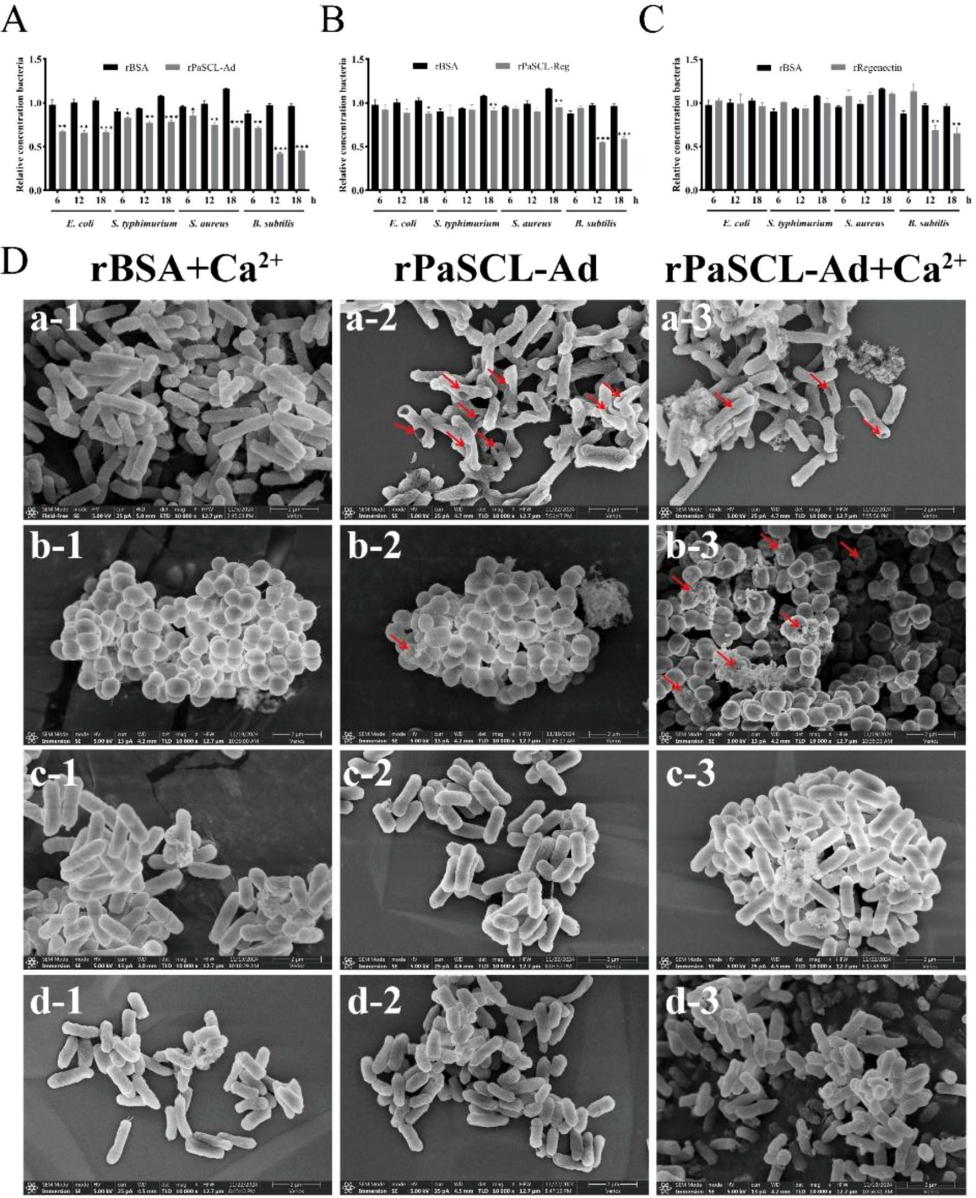
Direct bacteriostatic and bacteriocidal activities *in vitro* of rPaSCL-Ad, rPaSCL-Reg and rRegenectin. **(A)** The rPaSCL-Ad, rPaSCL-Reg and rRegenectin were incubated with *E. coli*, *S. aureus*, *S. typhimurium* and *B. subtilis* for 6 h, 12 h and 18 h Bacterial growth was assessed by measuring absorbance at 600 nm. rBSA protein was used as a negative control; **(B)** Scanning electron microscopy (SEM) was used to examine the observation on the interaction between rPaSCL-Ad and bacteria. Representative SEM images of *E. coli* (a), *S. aureus* (b), *S. typhimurium* (c), *B. subtilis* (d) are shown. Arrows indicate membrane pore formation. Asterisk indicates significant differences compared with values of the control (student’s t test, *p < 0.05, **p < 0.01, ***p < 0.001).

Following the observation that rPaSCL-Reg and rPaSCL-Ad bind to bacterial surfaces, their potential bacteriostatic or bactericidal activity was examined. Bacterial cultures exposed to intact rPaSCL-Reg, rPaSCL-Ad and rRegenectin at concentrations of 100 μg/ml showed significant variations in bacterial growth compared to control cultures with rBSA control group. The results revealed that only rPaSCL-Ad demonstrated direct bacteriostatic activity against all bacteria (**Figure 6A**), while rPaSCL-Reg and rRegenectin specifically inhibited *B. subtilis* growth (**Figure 6B** and C). Notably, scanning electron microscopy (SEM) analysis revealed that both GN *E. coli* and GP *S. aureus* bacteria exhibited membrane pore formation following rPaSCL-Ad treatment (**Figure 6B**). In contrast, rPaSCL-Reg did not compromise cell membrane integrity of GN or GP bacteria (**Supplementary Figure S3**).

### 3.6 rPaSCL-Ad and rPaSCL-Reg binds to bacterial surface components and to the Periplaneta americana hemocyte surface

**Figure 7:**
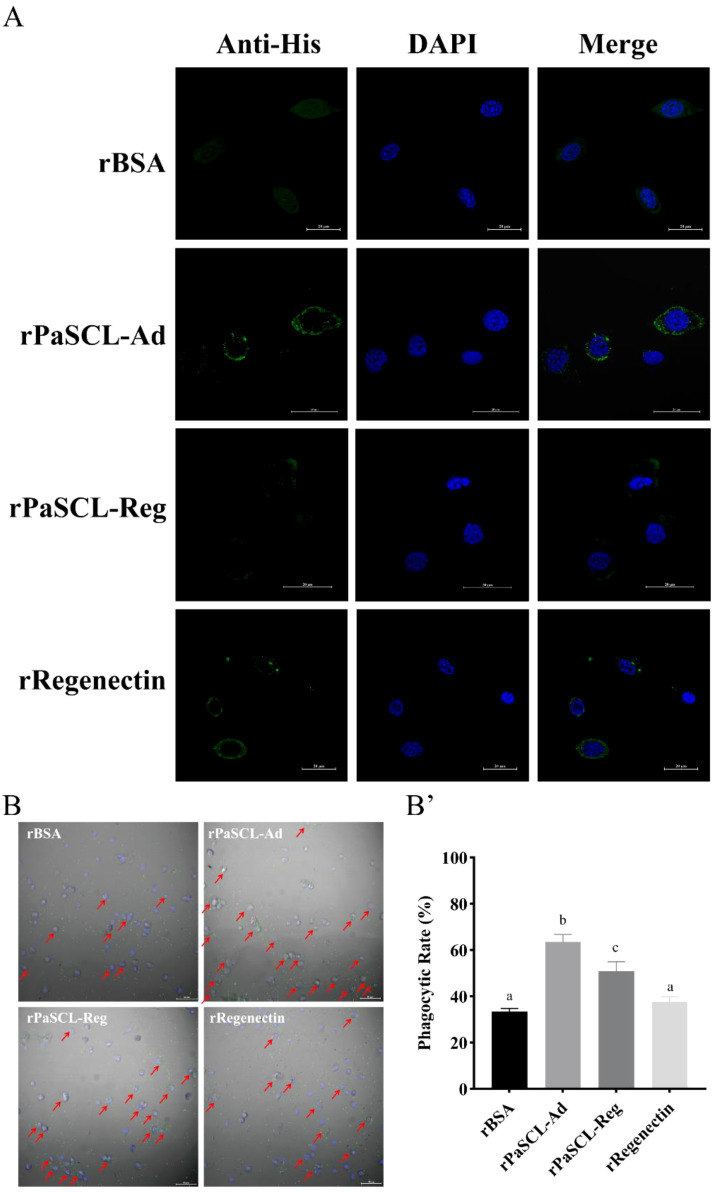
rPaSCL-Ad and rPaSCL-Reg bind to the hemocyte surface and enhance the phagocytic rate. **(A)** Immunocytochemical analysis was performed to examine the binding of rPaSCL-Ad and rPaSCL-Reg to the hemocyte surface. **(B)** Immunocytochemical assays were conducted to assess rPaSCL-Ad and rPaSCL-Reg enhanced phagocytosis *in vitro*. B’ The phagocytic rate was calculated after 2h, and statistical significance was analyzed using one-way ANOVA: *p < 0.05, **p < 0.01, ***p < 0.001. Arrows indicate sites of hemocyte phagocytic activity.

To determine whether rPaSCL-Ad, rPaSCL-Reg and rRegenectin are involved in hemocyte-mediated phagocytosis, their ability to bind to the hemocyte surface of *Periplaneta americana* was examined by immunocytochemical analysis. The results showed that all three recombinant proteins bound to the hemocyte surface (**Figure 7A**).

Subsequently, collected hemocytes were placed in cell culture plates and pre-incubated separately with each recombinant proteins, using rBSA as the control group. FITC-labeled *E. coli* was then added for co-incubation. After fixation, hemocytes phagocytosis of *E. coli* was visualized by microscopy and quantitatively analyzed. The results demonstrated that rPaSCL-Ad and rPaSCL-Reg significantly enhanced hemocyte phagocytosis of *E. coli* compared with the control, whereas rRegenectin had no detectable effect (**Figure 7B and B’**). These findings suggest that rPaSCL-Ad and rPaSCL-Reg promote hemocyte phagocytic activity by facilitating interactions between bacterial and hemocytes.

### 3.7 *PaSCLec* regulates the expression of antimicrobial peptides

**Figure 8:**
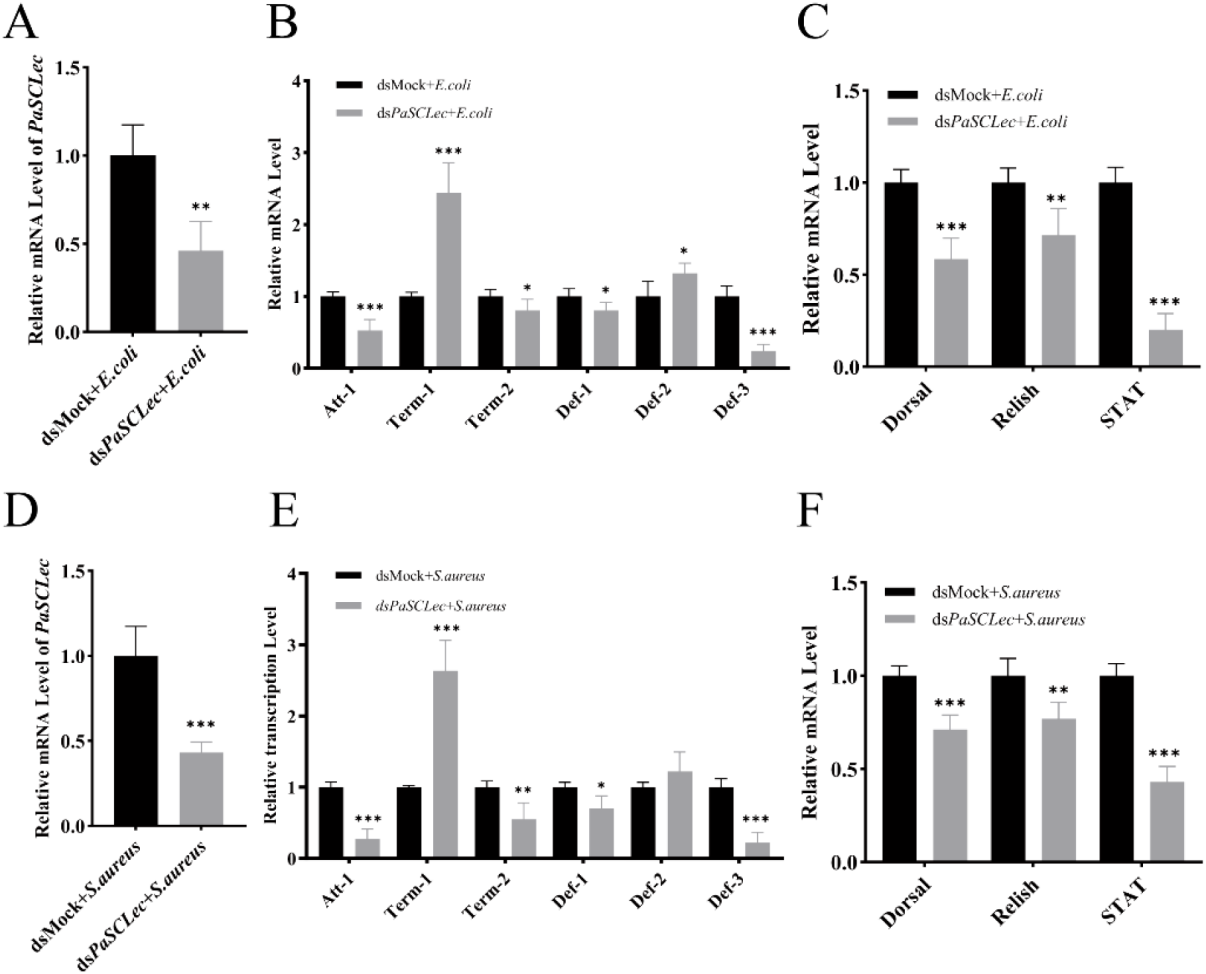
Expression of transcription factors and antimicrobial peptides (AMPs) following bacterial challenge after *PaSCLec* knockdown. Relative mRNA levels of *PaSCLec* following injection with *E. coli***(A)** or *S. aureus***(D)**. The mRNA expression levels of AMPs **(B, E)** and transcription factors **(C, F)** were measured after injection of *E. coli* and *S. aureus* at 24 h, respectively. Each group consisted of 15 larvae injected with dsRNA, and three biological replicates were performed. Asterisk indicates significant differences compared with values of the control (student’s t test, *p < 0.05, **p < 0.01, ***p < 0.001).

To investigate whether *PaSCLec* indirectly participates in immune responses through AMP expression regulation, ds*PaSCLec* was synthesized *in vitro* and injected into 6th *P. americana*. Following stimulation with *E. coli* and *S. aureus*, decreased relative mRNA levels of *PaSCLec* were observed (**Figures 8A, D**). Six AMPs were evaluated: *Attacin-1* (*Att-1*), *Termicin-1* (*Term-1*), *Termicin-2* (*Term-2*), *Defensin-1* (*Def-1*), *Defensin-2* (*Def-2*) and *Defensin-3* (*Def-3*). Bacterial challenges significantly downregulated the expression of antimicrobial peptides *Att-1*, *Term-2*, *Def-1* and *Def-3* (**Figures 8B, E**). Conversely, *Term-1* and *Def-2* expression were equally upregulated in the ds*PaSCLec* transfected cockroaches. To examine the potential role of *PaSCLec* in AMP regulation *in vivo*, its involvement in Toll, IMD, and JAK/STAT pathways was investigated via transcription factors *Dorsal*, *Relish*, and *STAT*. The results indicated significant reduction in *Dorsal*, *Relish* and *STAT* expression (**Figures 8C, F**).

**Figure 9:**
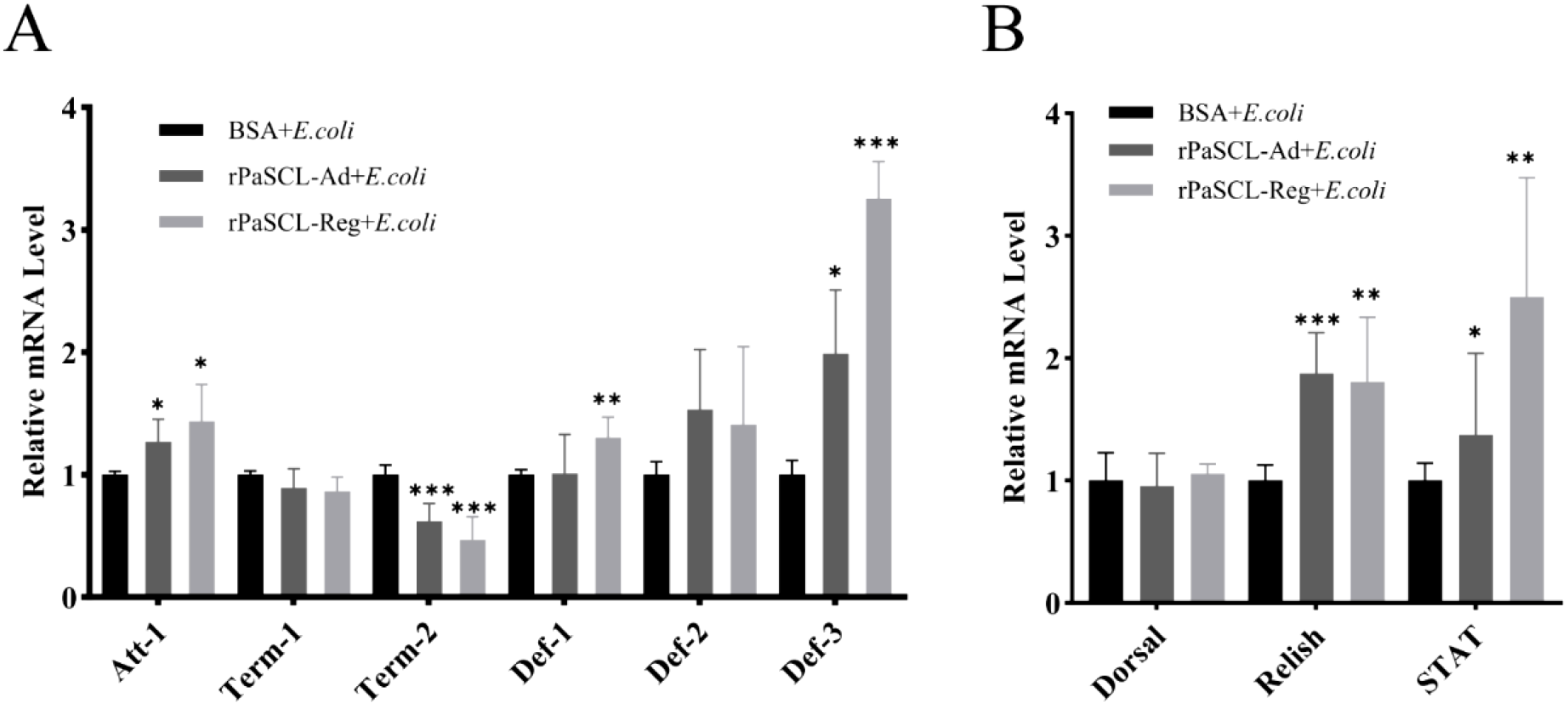
Expression of transcription factors and AMPs in rPaSCL-Ad-rescued and rPaSCL-Reg rescued cockroaches following *E. coli* challenge. The mRNA levels of AMPs **(A)** and transcription factors **(B)** were analyzed by qRT-PCR in rPaSCL-Ad- and rPaSCL-Reg-injected cockroaches. rBSA was used as the control. Asterisk indicates significant differences compared with values of the control (student’s t test, *p < 0.05, **p < 0.01, ***p < 0.001).

To validate these findings, ds*PaSCLec* transfected cockroaches were initially injected with rPaSCL-Ad and rPaSCL-Reg protein respectively, followed by *E. coli* challenge as previously described. The results demonstrated significantly increased Relish and STAT expression in response to *E. coli* challenge in both rPaSCL-Ad-rescued and rPaSCL-Reg-rescued cockroaches (**Figure 9A**). Additionally, the expression of *Att-1* and *Def-3*, along with transcription factors Relish and STAT, showed significant increases compared to control cockroaches (**Figure 9B**). These findings indicate that *PaSCLec* specifically upregulates the expression of *Att-1* and *Def-3*, suggesting that *PaSCLec* regulates AMP expression through multiple pathways.

### 3.8 *PaSCLec* is involved in reg regeneration of *P. americana*

**Figure 10:**
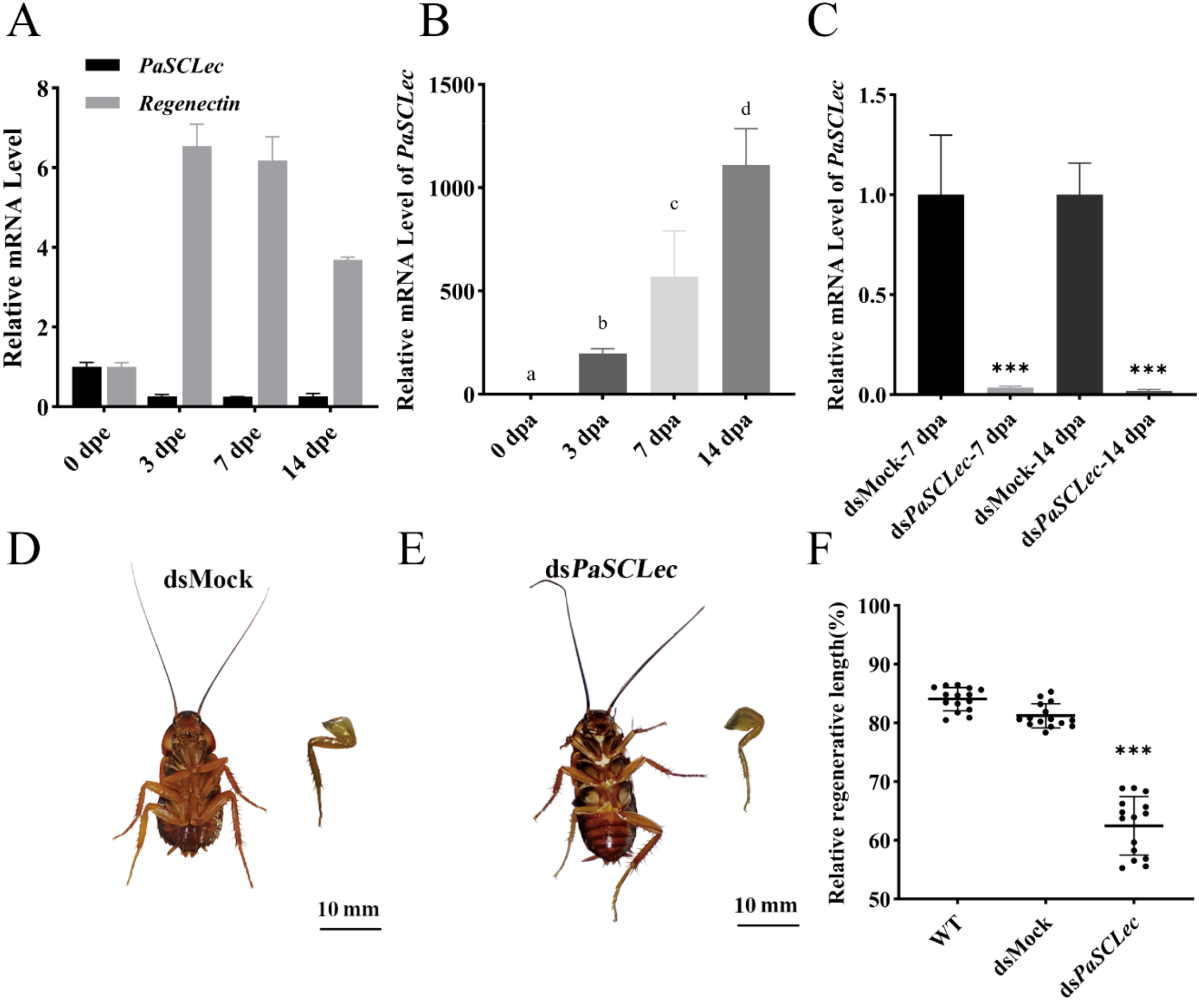
The C-type lectin *PaSCLec* regulates leg morphogenesis. **(A)** Relative mRNA level of *PaSCLec* and *Regenectin* during normal development. **(B)** Relative mRNA level of *PaSCLec* during leg regeneration. **(C)** Silencing efficiency of ds*PaSCLec* RNAi at 7 and 14 dpa. **(D)** Phenotype of regenerated legs in the dsMock-injected group. **(E)** Phenotype of regenerated legs in the ds*PaSCLec-*injected group. **(F)** Relative regenerative length of regenerated legs in Wide Type (WT), dsMock- and ds*PaSCLec-*treated groups. Each group including 15 larvae was injected into dsRNA. Different letters on the error bar indicate statistically significant differences at *p <* 0.05 level (ANOVA in association with Tukey’s HSD test). Asterisk indicates significant differences compared with values of the control (student’s t test, *p < 0.05, **p < 0.01, ***p < 0.001).

To investigate whether *PaSCLec* has an effect on leg regeneration, we first evaluated the expression of *PaSCLec* at 0, 3, 7 and 14 days post-ecdysis (dpe) or dpa. The results showed that the expression of *PaSCLec* was significantly upregulated during leg regeneration (**Figures 10A, B**). Then, RNAi of *PaSCLec* was performed and the interference efficiency of *PaSCLec* was measured on 7 dpa and 14 dpa, and the mRNA level of *PaSCLec* was significantly reduced compared with control (**Figure 10C**). Injection of ds*PaSCLec* caused the regenerated legs to exhibit morphological abnormalities, while no change was observed when dsMock was injected (**Figures 10D, E**). The regenerative length (Regenerated leg/Contralateral leg, %) was measured after RNAi treatments of genes (**Figure 10F**). In the wild-type (WT) group (N = 15), the mean relative regenerative length was 84.046% ± 1.98% (mean ± standard deviation, SD). The dsMock group, which served as a non-targeting control (N = 15), exhibited a similar relative regenerative length of 81.214% ± 2.06%. In contrast, the ds*PaSCLec* group, in which PaSCLec was knocked down (N = 15), showed a significantly reduced relative regenerative length of 62.473% ± 4.98%. Statistical analysis revealed that both WT and dsMock groups had markedly higher relative regenerative lengths compared to the ds*PaSCLec* group.

### 3.9 *PaSCLec* regulates the expression of AMPs during leg regeneration

**Figure 11:**
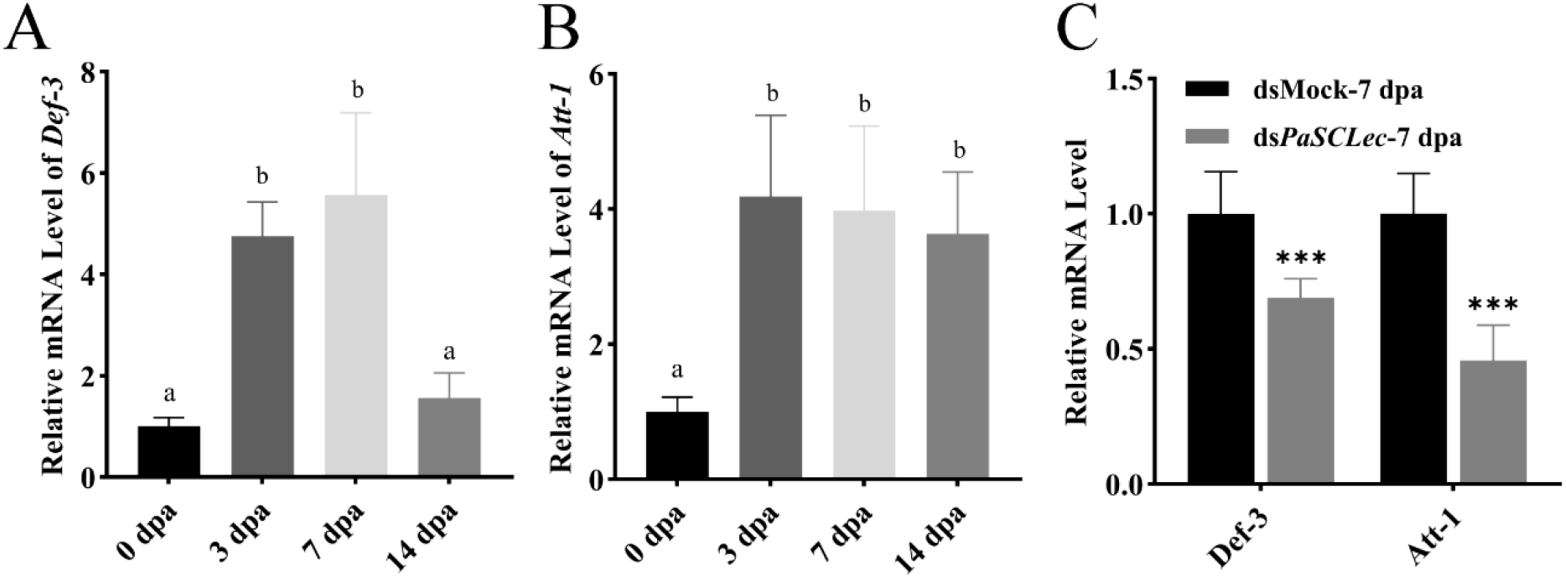
Effects of *PaSCLec* on *Def-3 and Att-1* during leg regeneration. **(A)** Relative mRNA level of *Def-3* during leg regeneration. **(B)** Relative mRNA level of *Att-1* during leg regeneration. **(C)** Relative mRNA level of *Def-3 and Att-1* determined by qRT-PCR following ds*PaSCLec* treatment. Each group consisted of 15 larvae injected with dsRNA. Different letters above the error bars indicate statistically significant differences at *p <* 0.05 level (ANOVA in association with Tukey’s HSD test). Asterisk indicates significant differences compared with values of the control (student’s t test, *p < 0.05, **p < 0.01, ***p < 0.001).

To investigate PaSCLec’s immune function during leg regeneration, we measured mRNA levels of *Def-3 and Att-1* regulated by *PaSCLec* in leg regeneration. Results indicated sustained expression of *Def-3 and Att-1* during early regeneration phases (**Figures 11A, B**), which were downregulated after injection of ds*PaSCLec* (**Figure 11C**).

### 3.10 *PaSCLec* and JAK/STAT signaling may synergistically regulate immunity and leg regeneration in *Periplaneta americana*

**Figure 12:**
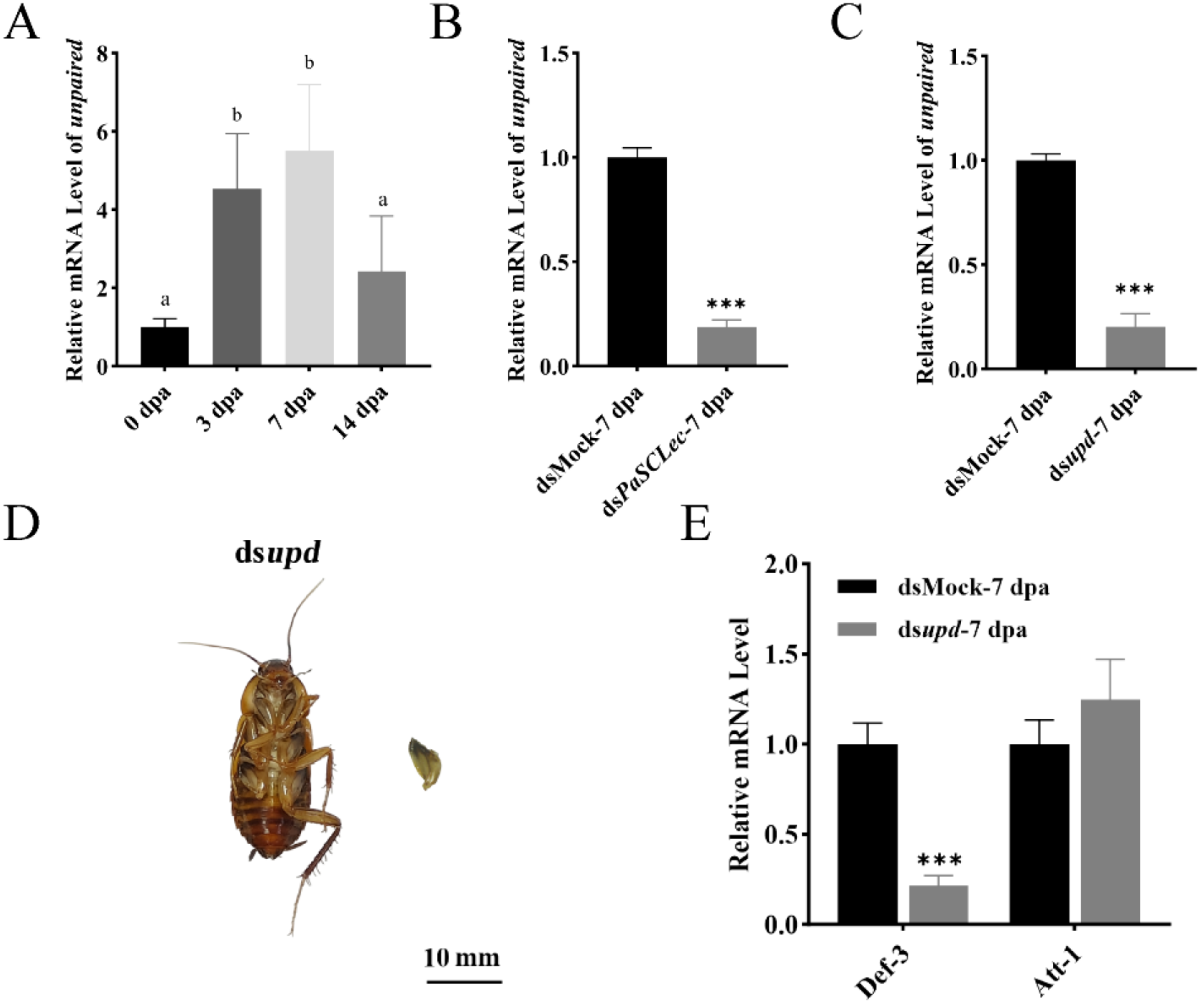
*PaSCLec* and *unpaired* regulate the *Def-3* expression and leg regeneration through a feedback loop. **(A)** Relative mRNA level of *unpaired* during leg regeneration. **(B)** Relative mRNA level of *unpaired* following *dsPaSCLec* treatment. **(C)** RNAi silencing efficiency of ds*upd* at 7 dpa. **(D)** RNAi treatment significantly disrupted the entire leg regeneration process. **(E)** Relative mRNA level of *PaSCLec*, *Def-3 and Att-1* following ds*upd* treatment. Each group consisted of 15 larvae injected with dsRNA. Different letters above the error bars indicate statistically significant differences at *p <* 0.05 level (ANOVA in association with Tukey’s HSD test). Asterisk indicates significant differences compared with values of the control (student’s t test, *p < 0.05, **p < 0.01, ***p < 0.001).

To explore the latent interaction of JAK/STAT signaling pathways with *PaSCLec* during leg regeneration, RNAi was performed to knockdown the expression of *PaSCLec* in *P. americana.* The expression of unpaired was upregulated at 3 dpa and 7 dpa and reduced significant at 7 dpa when the *PaSCLec* was silenced (**Figures 12A, B**). Our results showed that *PaSCLec* knockdown leads to reduced *unpaired* mRNA levels during leg regeneration, suggesting that *PaSCLec* may modulate the transcription of JAK/STAT associated cytokines. To confirm the role of the JAK/STAT pathway and specifically the functions of the ligand *unpaired*, we performed to knockdown the expression of *unpaired*. Silencing efficiency of RNAi was detected by on 7 dpa (**Figure 12C**). Knockdown of *unpaired* in the JAK/STAT signaling disrupted the entire regeneration process (**Figure 12D**) and the mRNA level of *Def-3* was significantly impaired (**Figure 12E**). In contrast, the mRNA level of *Att-1* was upregulated (**Figure 12E**). Because unpaired is a canonical activator of the JAK/STAT pathway, this correlation implies a possible functional relationship between *PaSCLec* and JAK/STAT-mediated processes in immunity and regeneration. Unpaired is a canonical activator of the JAK/STAT pathway, this correlation implies a possible functional relationship between *PaSCLec* and JAK/STAT mediated processes in immunity and regeneration. Nevertheless, the involvement of JAK/STAT signaling remains speculative, as the current study does not provide direct mechanistic evidence. We did not detect physical interactions, receptor activation, or downstream readouts such as STAT phosphorylation or nuclear translocation. Thus, additional experimental work will be required to define whether and how PaSCLec interacts with the JAK/STAT pathway in future.

## 4 Discussion

Secretory CTLs in the insect immune system are soluble PRRs that mediate innate immune defense by specifically recognizing evolutionarily conserved glycan motifs on pathogen surfaces (30, 31). In this study, we identified a novel secretory C-type lectin gene, *PaSCLec*, which has undergone evolutionary divergence through an alternative donor-site splicing event. This splicing event introduces a frameshift mutation that produces two distinct isoforms, PaSCL-Reg and PaSCL-Ad. Both PaSCL-Ad and PaSCL-Reg retain identical signal peptides and CLECT domains in their tertiary structures (**Figure 1**). However, the two isoforms exhibit functional divergence, including distinct ligand-binding specificities and differential regulatory effects on immune cell signaling pathways. A similar phenomenon has been reported in *Procambarus clarkii*, where the spliced isoform PcGal4-L-CRD exhibits stronger binding activity than PcGal4-L (32). These findings suggest that the strong upregulation of *PaSCLec* during *P. americana* leg regeneration contributes to tissue-specific immunoregulation and may be involved in pathogen recognition.

Tissue expression profiling revealed that *PaSCLec* is highly expressed in the hemolymph (**Figure 2A**), consistent with observations in other insect CTLs (33, 34). Previous studies indicate that secretory C-type lectins in the hemolymph modulate innate immunity through pathogen recognition (35) and antimicrobial peptide (AMP) induction (36), supporting the hypothesis that *PaSCLec* contributes to immune regulation. To test this hypothesis, Gram-negative *E. coli* and Gram-positive *S. aureus* were injected into *P. americana*. Following bacterial injection, *PaSCLec* expression in the hemolymph was first downregulated and subsequently upregulated (**Figures 2B, C**). A similar expression pattern has been reported in *Tribolium castaneum* and *Octodonta nipae* (7, 37). This response suggests that bacteria may transiently suppress CTL expression in the hemolymph as a strategy to evade host immune defenses (38). The decrease in *PaSCLec* mRNA levels after bacterial infection supports this possibility, although the underlying mechanism remains to be elucidated. Recognition and binding of bacterial carbohydrate structures, including LPS and PGN (39), likely underlie its agglutination activity (40). Accumulating evidence shows that CTLs display dose-dependent binding affinity toward both LPS and PGN (37, 41–43). The mutant rPaSCL-Ad exhibited significantly higher binding affinity for PAMPs than rPaSCL-Reg and rRegenectin (**Figures 4A–D**). These results indicate that although both PaSCLec and Regenectin are upregulated during limb regeneration, only the mutant rPaSCL-Ad shows strong *in vitro* binding to PAMPs, supporting its involvement in immune defense. Bacterial binding assays further confirmed the superior binding affinity of rPaSCL-Ad (**Figure 4E**), reinforcing this conclusion. Subsequent assays demonstrated that rPaSCL-Ad, rPaSCL-Reg, and rRegenectin agglutinate both GP and GN bacteria in a Ca²^+^-dependent manner, with rPaSCL-Ad displaying the strongest activity (**Figure 5**). This outcome is consistent with previous reports (44, 45).

Because both rPaSCL-Ad and rPaSCL-Reg bind and agglutinate bacteria, we next examined their bacteriostatic and bactericidal activities. Previous studies reported that rMaCTL inhibits the growth of multiple bacterial species *in vitro* (46). A similar inhibitory effect was observed for rPaSCL-Ad (**Figure 6A**). Similar effects were observed in the mutant rPaSCL-Ad (**Figure 6A**). SEM analysis showed that rPaSCL-Ad disrupted the cellular structures of *E. coli* and *S. aureus* (**Figure 6D**), potentially due to its strong binding to LPS and PGN. The truncated rPaSCL-Ad displayed marked antibacterial activity, whereas rPaSCL-Reg lacked detectable activity. Structural analysis suggests that the functional divergence arises from an exposed membrane-active segment in PaSCL-Ad that mediates membrane perforation. In contrast, the extended C-terminal region of PaSCL-Reg likely shields or neutralizes this segment. However, *S. typhimurium* and *B. subtilis* remained intact cellular structures. Both proteins may induce partial bacterial inactivation through agglutination-mediated mechanisms. Similar bactericidal activity exists in other CTLs. For example, the crab lectin EsIgLectin kills *V. parahaemolyticus* and *S. aureus* (47). The newly identified lectin Perlucin exhibits bactericidal activity against *V. parahaemolyticus* (48). The Pacific Abalone CTL shows broad-spectrum activity against both GP and GN bacteria (49).

The recombinant CTL enhances hemocyte phagocytic capacity by binding to hemocyte surface receptors or PAMPs. Previous research demonstrated that rTcCTL2 binds to microbes and promotes hemocyte-mediated phagocytosis *in vitro* (50). MjCC-CL binds to *E. coli* via recognition of cell-surface polysaccharides and also interacts with self-ligands on shrimp hemocyte surfaces (51). Similarly, rPaSCL-Ad and rPaSCL-Reg demonstrated binding to the hemocyte surface (**Figure 7A**), and *in vitro* experiments showed rPaSCL-Ad more effectively enhanced hemocyte phagocytic activity (**Figure 7B and B’**), suggesting their potential role as opsonins.

As PRRs, C-type lectins primarily recognize invading pathogens and trigger downstream immune responses. For example, CTL16 activates immune signaling pathways that promote AMP production (52). Knockdown of TcCTL15 has been shown to reduce the mRNA levels of *Dif*, *Rel*, and *STAT* (6). A novel chimeric CTL identified in *Marsupenaeus japonicus* induces the expression of specific AMPs through the JAK/STAT pathway (51). Following ds*PaSCLec* transfection in *P. americana*, subsequent challenges with *E. coli* and *S. aureus* resulted in the downregulation of four AMPs (*Att-1*, *Term-2*, *Def-1* and *Def-3*) and transcription factors *Dorsal*, *Relish*, and *STAT*. These results indicate that *PaSCLec* plays a regulatory role in the immune response (**Figure 8**).

To validate these findings, dsRNA-transfected *P. americana* were first injected with rPaSCL-Ad or rPaSCL-Reg proteins and then challenged with *E. coli* as described above. Although both rPaSCL-Ad and rPaSCL-Reg regulate AMP expression, their functions likely differ because rPaSCL-Ad contains a truncated structure. Specifically, rPaSCL-Ad appears to function primarily as an opsonin, whereas rPaSCL-Reg plays a major role in immune responses to microbial challenge (**Figure 9**). Recent studies have shown that dimerization of CLEC2D alters its ligand-binding properties and thereby modulates immune responses (53).A single carbohydrate-binding site per CML1 protomer was located at the dimer interface, where both protomers contribute to ligand engagement. This interaction results in a hexavalent lectin (54). These findings suggest that *PaSCLec* exerts antimicrobial activity by activating classical immune pathways, thereby selectively inducing AMP expression in response to *E. coli* and *S. aureus* challenge.

Cockroaches demonstrate superior regeneration capabilities within the class Insecta (23). qRT-PCR results revealed that *PaSCLec* was highly expressed during leg regeneration (**Figures 10A, B**), implying that *PaSCLec* possibly plays an important role in regulating leg regeneration of *P. americana.* Previous studies have shown that REG3A, a secreted C-type lectin of the regenerating (REG) family, promotes the growth of PDAC cell lines by binding directly to the extracellular domain of EGFR (55). In addition, CLEC3A activates the PI3K–AKT signaling pathway to enhance cell proliferation (14). RNAi experiments demonstrated that knockdown of *PaSCLec* caused distinct morphological abnormalities in regenerated legs (**Figures 10B–F**). These findings further indicate that *PaSCLec* plays an essential role in leg regeneration in *P. americana*.

Previous studies have shown that AMP expression is markedly upregulated at wound edges after skin injury, indicating that AMPs may act as wound-healing stimulators (56, 57). In addition, AMPs modulate host immune responses, thereby enhancing pathogen clearance and promoting tissue repair (58). In our study, the mRNA levels of *Att-1* and *Def-3* were upregulated during leg regeneration but were significant reduced in the ds*PaSCLec* group (**Figure 11**). These findings indicate that *PaSCLec* regulates the expression of *Att-1* and *Def-3* during bacterial immune responses and leg regeneration. However, the specific functions of these genes require further validation through *in vitro* and *in vivo* experiments as well as bioinformatic analyses.

We preliminarily investigated the mechanism by which *PaSCLec* influences leg regeneration. Previous studies showed that JAK/STAT signaling contributes to leg regeneration in *Gryllus bimaculatus* (59). Our results further demonstrate that *PaSCLec* may modulates immune responses through the JAK/STAT signaling pathway. The JAK/STAT pathway is an evolutionarily conserved signaling cascade that plays critical roles in development, physiological homeostasis, and regenerative responses following infection or tissue injury (60). Therefore, we examined the expression of *unpaired*, a ligand of the JAK/STAT pathway after knockdown of *PaSCLec*. The mRNA levels of *unpaired* were significantly reduced at 3 dpa and 7 dpa (**Figures 12A, B**), suggesting that *PaSCLec* may mediate the expression of *unpaired.*

Knockdown of *unpaired* significantly impaired the regeneration process (**Figures 12C, D**), consistent with previous research (20). Additionally, unpaired knockdown led to a significant decrease in the expression of *Def-3*(**Figure 12E**), indicating that *Def-3* is regulated by the JAK/STAT pathway. BmCTL5 has been proposed as an important PRR regulating the JAK/STAT pathway (61). In contrast, *Att-1* is likely regulated by other pathways, such as the Toll or Imd pathway (62), as its expression increased significantly after *unpaired* knockdown. Although knockdown of *PaSCLec* and *unpaired* caused morphological defects in regenerated legs, this study primarily relied on external phenotypic measurements to assess regenerative outcomes. In addition, JAK/STAT signaling pathway activity and the protein-level of *PaSCLec* were not determined. Consequently, our findings provide only a preliminary insight into the role of *PaSCLec* in regulating immunity and regeneration. Future studies incorporating histological examinations and functional assays will be necessary to comprehensively elucidate the mechanisms by which *PaSCLec* regulates regenerative processes.

In conclusion, we identified a novel secreted C-type lectin, *PaSCLec*, from *P. americana*. PaSCLec exhibited two major functions in *P. americana*. First, its two isoforms (PaSCL-Reg and PaSCL-Ad) participate in immune responses by mediating bacterial agglutination, exerting direct bacteriostatic activity, and regulating AMP expression to resist pathogenic invasion. Second, *PaSCLec* is highly expressed during leg regeneration. Knockdown of *PaSCLec* impairs this process and significantly reduces the mRNA levels of unpaired, suggesting that *PaSCLec* may regulate leg regeneration by modulating the JAK/STAT signaling pathway. Thus, additional experimental work will be required to define whether and how *PaSCLec* interacts with the JAK/STAT pathway in future. These finding expand our understanding of secreted C-type lectins and provide a theoretical foundation for elucidating their roles in regenerative processes.

## Data Availability

The original contributions presented in the study are included in the article/**Supplementary Material**. Further inquiries can be directed to the corresponding author.

## References

[1] Dodd RB Drickamer K . Lectin-like proteins in model organisms: implications for evolution of carbohydrate-binding activity. Glycobiology. (2001) 11:71R–9R. doi: 10.1093/glycob/11.5.71R, PMID: 11425795

[2] Kim T Ri S Ju K Shi W Zhou W Yu Y . A C-type lectin with a single carbohydrate-recognition domain (CRD) containing unique QPN/WDD motifs from Tegillarca granosa is involved in the innate immune defense. Fish Shellfish Immunol. (2023) 142:109093. doi: 10.1016/j.fsi.2023.109093, PMID: 37722437

[3] Cambi A Koopman M Figdor CG . How C-type lectins detect pathogens: C-type lectins and pathogens. Cell Microbiol. (2005) 7:481–8. doi: 10.1111/j.1462-5822.2005.00506.x, PMID: 15760448

[4] Li M Li C Ma C Li H Zuo H Weng S . Identification of a C-type lectin with antiviral and antibacterial activity from pacific white shrimp Litopenaeus vannamei. Dev Comp Immunol. (2014) 46:231–40. doi: 10.1016/j.dci.2014.04.014, PMID: 24792214

[5] Huang X Huang Y Shi Y-R Ren Q Wang W . Function of a novel C-type lectin with two CRD domains from Macrobrachium rosenbergii in innate immunity. Dev Comp Immunol. (2015) 49:121–6. doi: 10.1016/j.dci.2014.11.015, PMID: 25475962

[6] Wang S Ai H Zhang Y Bi J Gao H Chen P . Functional analysis of a multiple-domain CTL15 in the innate immunity, eclosion, and reproduction of tribolium castaneum. Cells. (2023) 12:608. doi: 10.3390/cells12040608, PMID: 36831275 PMC9954269

[7] Zhang Y Ai H Wang Y Zhang P Du L Wang J . A pattern recognition receptor C-type lectin *TcCTL14* contributes to immune response and development in the red flour beetle, *Tribolium castaneum*. Insect Sci. (2023) 30:1363–77. doi: 10.1111/1744-7917.13161, PMID: 36518010

[8] Chen P Ai H Liu Z Li C Li B . The dual functions of a newly identified C-type lectin (TcCTL17) in the immunity and development of *Tribolium castaneum*. Bull Entomol Res. (2025) 115:251–64. doi: 10.1017/S0007485324000920, PMID: 40099412

[9] Li C Ai H Zhang B Huang X Li B . C-type lectin 9 participates in the immune response, development and reproduction of Tribolium castaneum. Pesticide Biochem Physiol. (2025) 207:106223. doi: 10.1016/j.pestbp.2024.106223, PMID: 39672654

[10] Tanji T Ohashi-Kobayashi A Natori S . Participation of a galactose-specific C-type lectin in *Drosophila* immunity. Biochem J. (2006) 396:127–38. doi: 10.1042/BJ20051921, PMID: 16475980 PMC1450005

[11] Ao J Ling E Yu X-Q . Drosophila C-type lectins enhance cellular encapsulation. Mol Immunol. (2007) 44:2541–8. doi: 10.1016/j.molimm.2006.12.024, PMID: 17287021 PMC1876673

[12] Gong Y Xia Y Su Z Wang X Kou Y Ma B . A C-type lectin, rfCTL27, activates the immune defense in the red palm weevil rhynchophorus ferrugineus (A.G. Olivier 1791) (Coleoptera: curculionidae: dryophthorinae) by the recognition of gram-negative bacteria. Insects. (2024) 15:212. doi: 10.3390/insects15030212, PMID: 38535407 PMC10971258

[13] Song Z Tian M Dong Y Ren C Du Y Hu J . The C-type lectin IML-10 promotes hemocytic encapsulation by enhancing aggregation of hemocytes in the Asian corn borer Ostrinia furnacalis. Insect Biochem Mol Biol. (2020) 118:103314. doi: 10.1016/j.ibmb.2020.103314, PMID: 31926881

[14] Chen X Ji Y Feng F Liu Z Qian L Shen H . C-type lectin domain-containing protein CLEC3A regulates proliferation, regeneration and maintenance of nucleus pulposus cells. Cell Mol Life Sci. (2022) 79:435. doi: 10.1007/s00018-022-04477-x, PMID: 35864364 PMC11071857

[15] Wang J-X Cao B Ma N Wu K-Y Chen W-B Wu W . Collectin-11 promotes cancer cell proliferation and tumor growth. JCI Insight. (2023) 8. doi: 10.1172/jci.insight.159452, PMID: 36883567 PMC10077485

[16] Lee J-H . Invertebrate model organisms as a platform to investigate rare human neurological diseases. Exp Neurobiol. (2022) 31:1–16. doi: 10.5607/en22003, PMID: 35256540 PMC8907251

[17] Aidoo OF Osei-Owusu J Asante K Dofuor AK Boateng BO Debrah SK . Insects as food and medicine: a sustainable solution for global health and environmental challenges. Front Nutr. (2023) 10:1113219. doi: 10.3389/fnut.2023.1113219, PMID: 37388630 PMC10303143

[18] Chunduri JR Sagar SP . Insect brain proteomics: A case study of periplaneta americana. In: Islam William T , editor. Tissue Proteomics. Springer US, New York, NY (2025). p. 99–118. doi: 10.1007/978-1-0716-4298-6_8, PMID: 39716000

[19] Zeng C Liao Q Hu Y Shen Y Geng F Chen L . The role of periplaneta americana (Blattodea: blattidae) in modern versus traditional chinese medicine. J Med Entomol. (2019) 56:1522–6. doi: 10.1093/jme/tjz081, PMID: 31265723

[20] Li S Zhu S Jia Q Yuan D Ren C Li K . The genomic and functional landscapes of developmental plasticity in the American cockroach. Nat Commun. (2018) 9:1008. doi: 10.1038/s41467-018-03281-1, PMID: 29559629 PMC5861062

[21] Rao J Li H Zhang H Xiang X Ding X Li L . Periplaneta Americana (L.) extract activates the ERK/CREB/BDNF pathway to promote post-stroke neuroregeneration and recovery of neurological functions in rats. J Ethnopharmacol. (2024) 321:117400. doi: 10.1016/j.jep.2023.117400, PMID: 37952730

[22] Yang Y Huang J Li X Lin R Wang X Xiao G . Periplaneta americana extract promotes infectious diabetic ulcers wound healing by downregulation of LINC01133/SLAMF9. Chin J Natural Medicines. (2024) 22:608–18. doi: 10.1016/S1875-5364(24)60569-8, PMID: 39059830

[23] Zhong J Jing A Zheng S Li S Zhang X Ren C . Physiological and molecular mechanisms of insect appendage regeneration. Cell Regener. (2023) 12:9. doi: 10.1186/s13619-022-00156-1, PMID: 36859631 PMC9978051

[24] Kubo T Natori S . Purification and some properties of a lectin from the hemolymph of Periplaneta americana (American cockroach). Eur J Biochem. (1987) 168:75–82. doi: 10.1111/j.1432-1033.1987.tb13389.x, PMID: 3665920

[25] Kawasaki K Kubo T Natori S . A novel role of Periplaneta lectin as an opsonin to recognize 2-Keto-3-deoxy octonate residues of bacterial lipopolysaccharides. Comp Biochem Physiol Part B: Comp Biochem. (1993) 106:675–80. doi: 10.1016/0305-0491(93)90148-X, PMID: 8281762

[26] Kawasaki K Kubo T Natori S . Presence of the periplaneta lectin-related protein family in the american cockroach Periplaneta americana. Insect Biochem Mol Biol. (1996) 26:355–64. doi: 10.1016/0965-1748(95)00101-8, PMID: 8814782

[27] Kubo T Kawasaki K Natori S . Transient appearance and localization of a 26-kDa lectin, a novel member of the periplaneta lectin family, in regenerating cockroach leg. Dev Biol. (1993) 156:381–90. doi: 10.1006/dbio.1993.1085, PMID: 8462738

[28] Liu X Sun N Wu X Wu J Xian S Wang D . Comparative transcriptome analysis reveals epithelial growth factor receptor (EGFR) pathway and secreted C-type lectins as essential drivers of leg regeneration in periplaneta americana. Insects. (2025) 16:934. doi: 10.3390/insects16090934, PMID: 41009115 PMC12470663

[29] Pfaffl MW . A new mathematical model for relative quantification in real-time RT-PCR. Nucleic Acids Res. (2001) 29:45e–45. doi: 10.1093/nar/29.9.e45, PMID: 11328886 PMC55695

[30] Zhu Y Yu X Cheng G . Insect C-type lectins in microbial infections. In: Hsieh S-L , editor. Lectin in Host Defense Against Microbial Infections. Springer Singapore, Singapore (2020). p. 129–40. doi: 10.1007/978-981-15-1580-4_5, PMID: 32152945

[31] Chen H-Y Li W-Y Wang J Bo G-W Yang G-W Yang H-T . A C-type lectin containing two carbohydrate recognition domains participates in the antibacterial response by regulating the JNK pathway and promoting phagocytosis. Fish Shellfish Immunol. (2022) 127:349–56. doi: 10.1016/j.fsi.2022.06.007, PMID: 35752372

[32] Luo T Ren X Fan L Guo C Zhang B Bi J . Identification of two galectin-4 proteins (PcGal4-L and PcGal4-L-CRD) and their function in AMP expression in Procambarus clarkii. Fish Shellfish Immunol. (2023) 141:109040. doi: 10.1016/j.fsi.2023.109040, PMID: 37648118

[33] Wang P Zhuo X-R Tang L Liu X-S Wang Y-F Wang G-X . C-type lectin interacting with β-integrin enhances hemocytic encapsulation in the cotton bollworm, Helicoverpa armigera. Insect Biochem Mol Biol. (2017) 86:29–40. doi: 10.1016/j.ibmb.2017.05.005, PMID: 28572000

[34] Sun Y-X Zhang B-X Liu F-F Rao X-J . Functional characterization of *Bombyx mori* (Lepidoptera: Bombycidae) C-type lectin 5. J Econ Entomol. (2023) 116:1862–75. doi: 10.1093/jee/toad142, PMID: 37540584

[35] Mei X Li C Peng P Wang J He E Qiu Z . Bombyx mori C-Type Lectin (BmIML-2) Inhibits the Proliferation of B. mori Nucleopolyhedrovirus (BmNPV) through Involvement in Apoptosis. IJMS. (2022) 23:8369. doi: 10.3390/ijms23158369, PMID: 35955502 PMC9369074

[36] Wang G-J Wang J-L Liu X-S . Identification and analysis of C-type lectins from Helicoverpa armigera in response to the entomopathogenic fungus Metarhizium rileyi infection. Dev Comp Immunol. (2023) 140:104620. doi: 10.1016/j.dci.2022.104620, PMID: 36528221

[37] Zhang H-J Lin Y-P Liu M Liang X-Y Ji Y-N Tang B-Z . Functional conservation and division of two single-carbohydrate-recognition domain C-type lectins from the nipa palm hispid beetle Octodonta nipae (Maulik). Dev Comp Immunol. (2019) 100:103416. doi: 10.1016/j.dci.2019.103416, PMID: 31255631

[38] Eddie Ip WK Takahashi K Alan Ezekowitz R Stuart LM . Mannose-binding lectin and innate immunity. Immunol Rev. (2009) 230:9–21. doi: 10.1111/j.1600-065X.2009.00789.x, PMID: 19594626

[39] Xia X You M Rao X-J Yu X-Q . Insect C-type lectins in innate immunity. Dev Comp Immunol. (2018) 83:70–9. doi: 10.1016/j.dci.2017.11.020, PMID: 29198776

[40] Ming Z Chen Z Tong H Zhou X Feng T Dai J . Immune functions of C-type lectins in medical arthropods. Insect Sci. (2024) 31:652–62. doi: 10.1111/1744-7917.13169, PMID: 36661334

[41] Gardos G Cole JO . Maintenance antipsychotic therapy: is the cure worse than the disease? Am J Psychiatry. (1976) 133:32–6. doi: 10.1176/ajp.133.1.32, PMID: 2021

[42] Wang J Guo X-L Chen H-Y Xiao L-X Yang G-W Yang H-T . A novel l-rhamnose-binding lectin participates in defending against bacterial infection in zebrafish. Fish Shellfish Immunol. (2023) 134:108553. doi: 10.1016/j.fsi.2023.108553, PMID: 36693487

[43] Ning M Li Q Fan L Guo C Zhang B Li J . RNA interference-mediated silencing of ctl13 inhibits innate immunity and development in stored pest Tribolium castaneum. Pesticide Biochem Physiol. (2024) 204:106104. doi: 10.1016/j.pestbp.2024.106104, PMID: 39277426

[44] Zhang L Wei C Guo Y Hu J Wang M . Molecular identification and functional characterization of a C-type lectin gene in Meretrix meretrix. Fish Shellfish Immunol. (2024) 153:109833. doi: 10.1016/j.fsi.2024.109833, PMID: 39147178

[45] Li E Ji J Kong W Shen D Li C An C . A C-type lectin with dual carbohydrate recognition domains functions in innate immune response in Asian corn borer, *Ostrinia furnacalis*. Insect Sci. (2025) 32:172–92. doi: 10.1111/1744-7917.13382, PMID: 38772748

[46] Liu Y Wang Z Wang W Liu B Li C Sun Y . Characterization and functional analysis of a novel C-type lectin in blunt snout bream (Megalobrama amblycephala). Fish Shellfish Immunol. (2023) 140:108966. doi: 10.1016/j.fsi.2023.108966, PMID: 37482206

[47] Zhou K Qin Y Song Y Zhao K Pan W Nan X . A novel ig domain–containing C-type lectin triggers the intestine–hemocyte axis to regulate antibacterial immunity in crab. J Immunol. (2022) 208:2343–62. doi: 10.4049/jimmunol.2101027, PMID: 35508356

[48] Wang Z Yang L Zhao Z Weng S He J Xu X . A novel perlucin with immune regulatory functions protects Litopenaeus vannamei against Vibrio parahaemolyticus infection. Fish Shellfish Immunol. (2024) 155:110028. doi: 10.1016/j.fsi.2024.110028, PMID: 39557373

[49] Choi M-J Kim YR Park NG Kim C-H Oh YD Lim HK . Characterization of a C-Type Lectin Domain-Containing Protein with Antibacterial Activity from Pacific Abalone (Haliotis discus hannai). IJMS. (2022) 23:698. doi: 10.3390/ijms23020698, PMID: 35054883 PMC8775961

[50] Wang S Miao S Lu Y Li C Li B . A C-type lectin (CTL2) mediated both humoral and cellular immunity against bacterial infection in Tribolium castaneum. Pesticide Biochem Physiol. (2024) 201:105852. doi: 10.1016/j.pestbp.2024.105852, PMID: 38685211

[51] Sun J-J Lan J-F Zhao X-F Vasta GR Wang J-X . Binding of a C-type lectin’s coiled-coil domain to the Domeless receptor directly activates the JAK/STAT pathway in the shrimp immune response to bacterial infection. PloS Pathog. (2017) 13:e1006626. doi: 10.1371/journal.ppat.1006626, PMID: 28931061 PMC5645147

[52] Bi J Wang Y Gao R Liu P Jiang Y Gao L . Functional analysis of a CTL-X-type lectin CTL16 in development and innate immunity of tribolium castaneum. IJMS. (2023) 24:10700. doi: 10.3390/ijms241310700, PMID: 37445878 PMC10341621

[53] Li F Wang H Li Y-Q Gu Y Jia X-M . C-type lectin receptor 2d forms homodimers and heterodimers with TLR2 to negatively regulate IRF5-mediated antifungal immunity. Nat Commun. (2023) 14:6718. doi: 10.1038/s41467-023-42216-3, PMID: 37872182 PMC10593818

[54] Bleuler-Martinez S Varrot A Olieric V Schubert M Vogt E Fetz C . Structure–function relationship of a novel fucoside-binding fruiting body lectin from *Coprinopsis cinerea* exhibiting nematotoxic activity. Glycobiology. (2022) 32:600–15. doi: 10.1093/glycob/cwac020, PMID: 35323921 PMC9191617

[55] Ren X Teng Y Xie K He X Chen G Zhang K . REG3A secreted by peritumoral acinar cells enhances pancreatic ductal adenocarcinoma progression via activation of EGFR signaling. Cell Commun Signal. (2025) 23:96. doi: 10.1186/s12964-025-02103-4, PMID: 39966859 PMC11837727

[56] Braff MH Bardan A Nizet V Gallo RL . Cutaneous defense mechanisms by antimicrobial peptides. J Invest Dermatol. (2005) 125:9–13. doi: 10.1111/j.0022-202x.2004.23587.x, PMID: 15982297

[57] Mahlapuu M Håkansson J Ringstad L Björn C . Antimicrobial peptides: an emerging category of therapeutic agents. Front Cell Infect Microbiol. (2016) 6:194. doi: 10.3389/fcimb.2016.00194, PMID: 28083516 PMC5186781

[58] Haidari H Melguizo-Rodríguez L Cowin AJ Kopecki Z . Therapeutic potential of antimicrobial peptides for treatment of wound infection. Am J Physiology-Cell Physiol. (2023) 324:C29–38. doi: 10.1152/ajpcell.00080.2022, PMID: 36409176

[59] Bando T Ishimaru Y Kida T Hamada Y Matsuoka Y Nakamura T . Analysis of RNA-Seq data reveals involvement of JAK/STAT signalling during leg regeneration in the cricket *Gryllus bimaculatus*. Development. (2013) 140:959–64. doi: 10.1242/dev.084590, PMID: 23344706

[60] Housden BE Perrimon N . Spatial and temporal organization of signaling pathways. Trends Biochem Sci. (2014) 39:457–64. doi: 10.1016/j.tibs.2014.07.008, PMID: 25155749 PMC4477539

[61] Geng T Lu F Wu H Wang Y Lou D Tu N . C-type lectin 5, a novel pattern recognition receptor for the JAK/STAT signaling pathway in Bombyx mori. J Invertebrate Pathol. (2021) 179:107473. doi: 10.1016/j.jip.2020.107473, PMID: 32946913

[62] Tanji T Hu X Weber ANR Ip YT . Toll and IMD pathways synergistically activate an innate immune response in *drosophila melanogaster*. Mol Cell Biol. (2007) 27:4578–88. doi: 10.1128/mcb.01814-06, PMID: 17438142 PMC1900069

