# Supplementary material for: A secreted C-type lectin in Periplaneta americana functioning in antibacterial activity, innate immune signaling and leg regeneration
Source: Front Immunol. 2026 Jan 20;16:1730116. doi: 10.3389/fimmu.2025.1730116 (PMC12864434; doi:10.3389/fimmu.2025.1730116)
Supplement: Supplementary file 1 [file DataSheet1.docx]

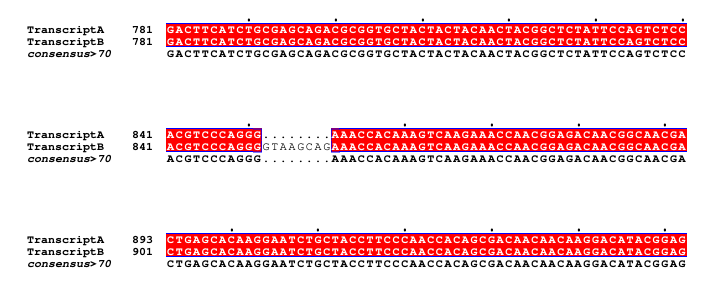


Fig. S1 Two transcript sequences alignment of *PaSCLec*.


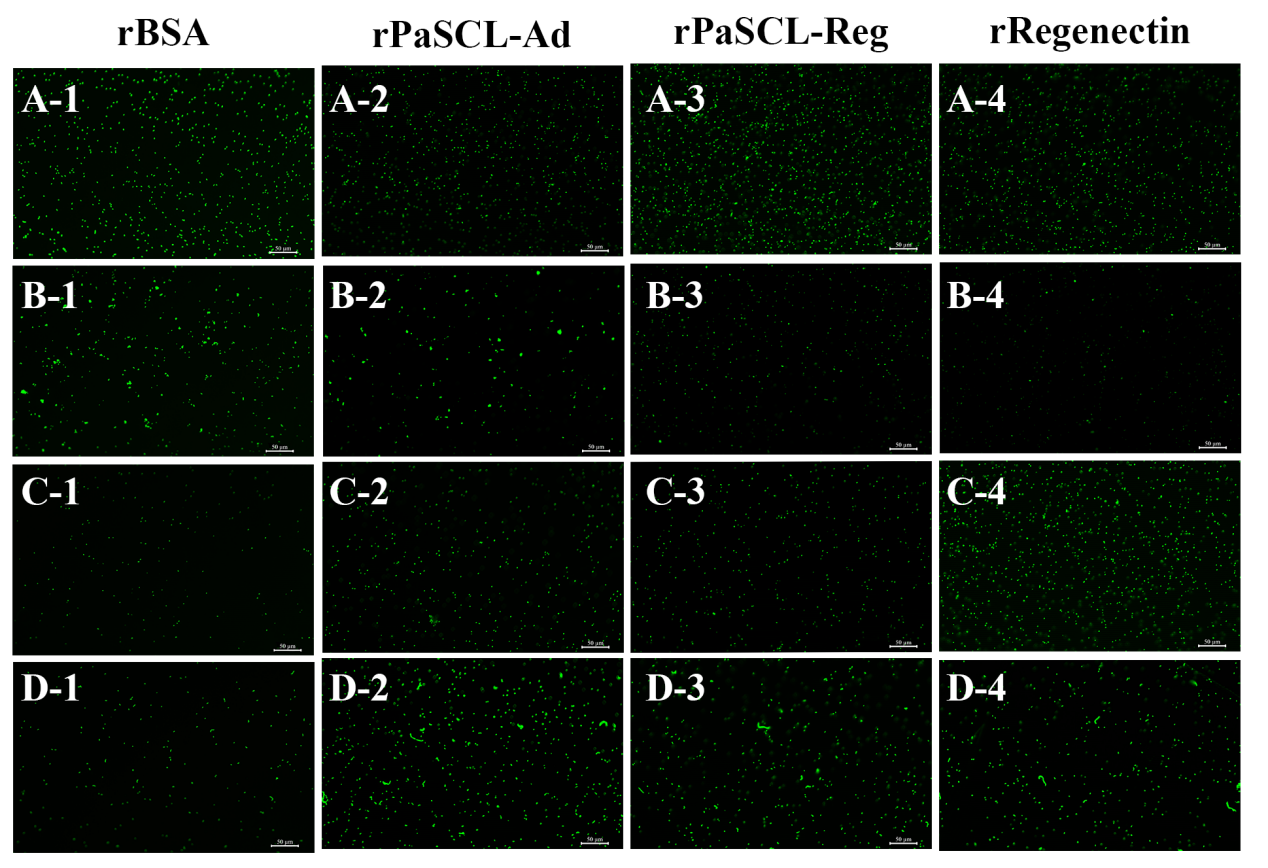


Fig. S2 Agglutination activities of rBSA and rPaSCL-Ad, rPaSCL-Reg and rRegenectin without Ca^2+^.

^
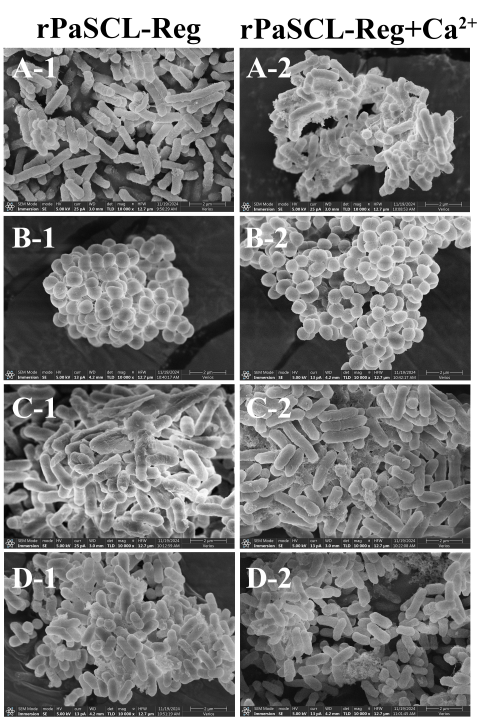
^

Fig. S3 SEM observation on the interaction between rPaSCL-Reg and bacteria. *E. coli* (A), *S. aureus* (B), *S. typhimurium* (C), *B. subtilis* (D).
